# Inhibitors of Bcl-2 and Bruton’s tyrosine kinase synergize to abrogate diffuse large B-cell lymphoma growth in vitro and in orthotopic xenotransplantation models

**DOI:** 10.1038/s41375-021-01470-4

**Published:** 2021-11-18

**Authors:** Katrin Bertram, Peter John Leary, Christophe Boudesco, Jonas Fullin, Kristin Stirm, Vineet Dalal, Thorsten Zenz, Alexandar Tzankov, Anne Müller

**Affiliations:** 1 Institute of Molecular Cancer Research and University of Zurich, Zurich, Switzerland; 2 Functional Genomics Center Zurich, University of Zurich, Zurich, Switzerland; 3 German Cancer Research Center, Heidelberg, Germany; 4 Comprehensive Cancer Center Zurich, Zurich, Switzerland; 5 Department of Medical Oncology and Hematology, University Hospital Zurich and University of Zurich, Zurich, Switzerland; 6 Institute of Medical Genetics and Pathology, University Hospital Basel, University of Basel, Basel, Switzerland

**Keywords:** Preclinical research, B-cell lymphoma, Translational research

## Abstract

Numerous targeted therapies have been developed for diffuse large B-cell lymphoma, but the results of late-stage clinical trials were mostly disappointing and have led to very few new regulatory approvals. Here, we use single and combinatorial drug response profiling to show that the combined inhibition of the anti-apoptotic protein Bcl-2 and of the tyrosine kinase BTK with the small molecules venetoclax and ibrutinib efficiently kills DLBCL cells in vitro. High Bcl-2 expression due to either *BCL2* amplifications or translocations, in conjunction with chronic active BCR signaling accurately predict responses to dual Bcl-2/BTK inhibition. Orthotopic xenotransplantation and patient-derived xenograft models confirm that the combinatorial is superior to single-agent treatment in reducing the lymphoma burden. Combinatorial treatment further efficiently overcomes both primary and acquired resistance to venetoclax, which we could link to reduced expression of the Bcl-2 family members Bcl-X_L_ and Bcl-2A1 under ibrutinib. We found in a Swiss DLBCL cohort that ~15% of patients are projected to respond to the venetoclax/ibrutinib combination based on their high Bcl-2 expression and nuclear NF-κB localization. Our data show that drug sensitivities exposed by drug response profiling can be attributed to specific mutational signatures and immunohistochemical biomarkers, and point to combined Bcl-2/BTK inhibition as a promising therapeutic strategy in DLBCL.

## Introduction

Diffuse large B-cell lymphoma (DLBCL) is an aggressive malignancy of the mature B-cell that may arise *de novo* in both lymphoid and non-lymphoid organs. DLBCL remains fatal in over a third of patients; the one major step forward has been the addition of the CD20-specific antibody rituximab to standard chemotherapy almost two decades ago [1, 2]. CAR T-cell therapy is available for a very select group of DLBCL patients only [3] and the CD79B-directed antibody-drug conjugate polatuzumab vedotin was recently approved for another highly select subgroup of relapsed/refractory DLBCL patients after at least two prior therapies [4, 5]. DLBCL originates from antigen-exposed B-cells that have undergone the germinal center (GC) reaction [6]. One of the hallmarks of the disease is its intertumoral heterogeneity [7]. Whereas gene expression profiling has traditionally been used to identify two major subsets of DLBCL that differ in their cell of origin, i.e., the activated B-cell and GC B-cell subtypes (ABC- and GCB-DLBCL) [8, 9], more recent studies have distinguished up to four [10] or five [11] different molecular subtypes based on transcriptional and mutational signatures, somatic copy number alterations and structural variants. These subtypes differ strongly in their response to standard of care treatments and survival probability. In particular, the refined stratification based on genetic abnormalities has led to the differentiation of two subtypes of ABC-DLBCL, of which one is characterized by (co-occurring) *MYD88* and *CD79B* mutations and *BCL2* gains, extranodal manifestations, a genetic signature of aberrant somatic hypermutation driven by activation-induced cytidine deaminase activity and a dismal prognosis; the other subtype is characterized by *NOTCH2* and *BCL6* mutations and structural aberrations, respectively, and the associated downstream transcriptional signatures, a presumably extrafollicular origin more reminiscent of marginal zone lymphoma, and a comparatively superior prognosis [10, 11]. Similarly, GCB-DLBCL can be stratified into two subtypes, of which one typically harbors translocations juxtaposing *BCL2* to the *IgH* enhancer in combination with frequent mutations in the chromatin modifers *KMT2D*, *CREBBP,* and *EZH2*, and inactivating *PTEN* mutations, bears similarities to the genetic landscape of follicular lymphoma and features a poor prognosis, whereas the other is a relatively low-risk subtype with mutations in PI3K, JAK/STAT, and BRAF pathway components [10, 11].

The substantial intertumoral heterogeneity of DLBCL suggests that individual tumors will exhibit vastly different susceptibilities to drugs- both approved compounds and drugs in clinical development. Surprisingly little attention has been paid in the field to differential drug responses in DLBCL and to the possible use of drug response profiling as a tool for guiding treatment decisions. Here, we report on a screen conducted on 19 DLBCL and other lymphoma cell lines, of drug susceptibility to a selection of 126 compounds that are approved for clinical use. Our results suggest that the Bcl-2 inhibitor venetoclax, in combination with the BTK inhibitor ibrutinib, is highly effective in the treatment of a particularly aggressive DLBCL subtype exhibiting *BCL2* amplifications on the one hand, and a constitutively active B-cell receptor/Bruton’s tyrosine kinase/NF-κB signaling axis on the other.

## Methods

### Cell culture experimentation, drug screening, and viability assays

We used a panel of 13 previously described DLBCL [12–14], four Burkitt lymphoma and three mantle cell lymphoma cell lines that are described in detail in the supplementary methods with respect to their culture conditions and authentication. Apoptosis rates were determined by TMRE staining and cell viability was assessed using the CellTiter-Blue metabolic activity assay (Promega). Drug screening conditions, procedures for lentiviral gene transfer and genomic editing, Western blotting, qRT-PCR and immunohistochemistry, RNAseq data analysis, and the Cancer Genome Atlas (TCGA) data analysis are all described in the supplementary methods.

### Animal experimentation and tissue processing

M-CSF^h^;IL-3/GM-CSF^h^;hSIRPA^tg^;TPO^h^;Rag2^−^γc^−^ (MISTRG) [15] and MISTRG mice that additionally express IL-6^h^ (MISTRG6) [16] were obtained from a local repository. For the orthotopic xenotransplantation model, cells were injected intravenously (1 × 10^7^ cells in 100 µl PBS) into 6- to 8-week-old mixed-gender MISTRG or MISTRG6 mice. Spleens and bone marrow from hind legs were harvested for analysis at the study endpoint of orthotopic models. All animal studies were reviewed and approved by the Zurich Cantonal Veterinary Office (licenses 224/2014, 235/2015, 132/2019 and their amendments, to AM). For patient‐derived xenograft transplantation, primary DLBCL cells (obtained from the Clinic of Hematology–Oncology at the University of Zürich) were transplanted by i.v. injection (1 × 10^6^ cells) to MISTRG6 mice as previously described [12]. Ethical approval for work with primary DLBCL cells was obtained from the Ethical Commission of the Canton of Zurich (KEK-ZH-Nr. 2009-0062/1). Drug administration routes and dosing, mouse randomization, and group sizes are all explained in detail in the supplementary methods section.

### Statistics

All statistical analyses were performed using GraphPad Prism software. Graphs represent means plus standard deviation of at least two independent experiments, and statistical analysis was performed using two-tailed Mann–Whitney test, or one-way ANOVA (in the case of normal data distribution) or by non-parametric ANOVA (Kruskal–Wallis test, in the case of non-normal data distribution) with Tukey’s multiple comparisons correction. For animal experiments, studies were repeated and multiple studies were pooled where one experiment alone was not sufficient to reach statistically significant conclusions.

## Results

### Drug response profiling identifies specific sensitivities of DLBCL cell lines that can be confirmed by genetic ablation of the drug target

**Fig. 1:**
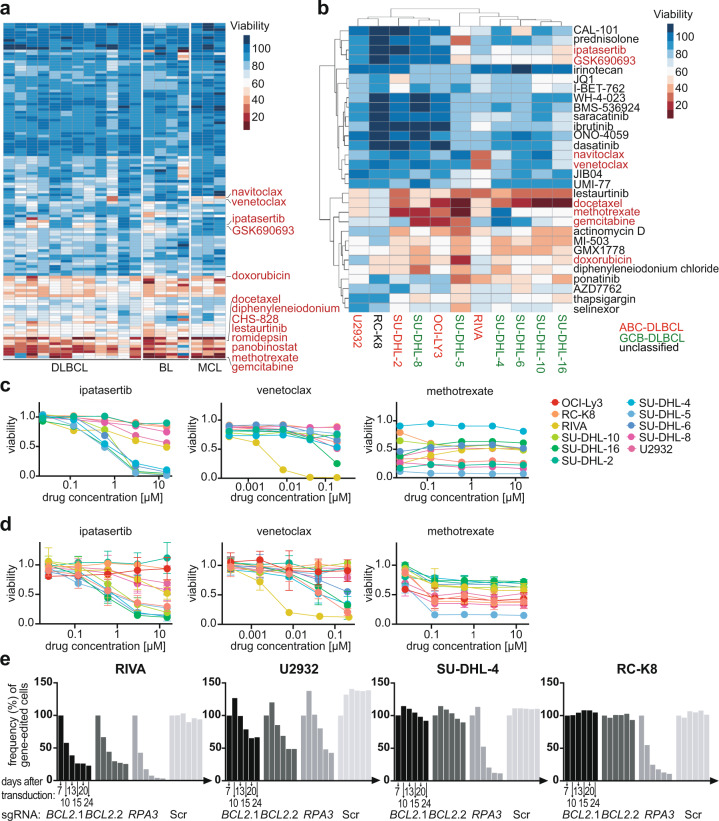
Drug response profiling exposes differential dependencies of DLBCL and other lymphoma cell lines on pathways that are commonly deregulated in hematological malignancies. **a** Heat map displaying the viability (calculated as the mean of the five concentrations assessed per drug) of 19 cell lines of the indicated entities, as assessed by CellTiter-Glo viability assay, after 48 h of exposure to 126 manually selected compounds targeting deregulated pathways in hematological malignancies. Select compounds with differential effects on viability are indicated. Five concentrations, generated by serial (fivefold) dilution, were tested per compound. See Supplementary Table 1 for the compounds and their respective highest concentrations. MCL, mantle cell lymphoma; BL, Burkitt lymphoma. Select compounds are indicated in red. **b** Heat map showing the viability of the subgroup of DLBCL cell lines only, and their differential responses to the indicated compounds. Cell lines are color-coded based on cell-of-origin (ABC-DLBCL in red, GCB-DLBCL in green, unclassified in black). **c** Viability curves, derived from the drug screen, of the indicated cell lines, showing all five assessed concentrations and three drugs targeting AKT signaling (ipatasertib), Bcl-2 (venetoclax), and dihydrofolate reductase (methotrexate). **d** Validation of viability after 48 h of exposure to the same three compounds, as assessed individually by CellTiter-Blue assay. Means ± standard deviations are shown for two to three independent experiments per cell line and drug, each quantified in duplicate measurements. **e** Competitive proliferation assay, performed over a period of 24 days, of the four indicated cell lines subjected to CRISPR-based editing of the *BCL2* locus, the *RPA* locus as positive control representing an essential gene, and a control guide RNA. Successfully targeted cells were identified by their blue fluorescent protein (BFP) expression and mixed 1:1 with non-edited cells at the start of the experiment. Two different guide RNAs are shown for Bcl-2. Data in **e** are representative of three independent experiments.

We systematically assessed the sensitivities of 19 lymphoma cell lines (mantle cell, Burkitt and DLBCL) to 126 FDA-approved compounds, which were chosen based on their activity on pathways that are known to be deregulated in hematopoietic malignancies (Supplementary Table 1). Cells were exposed to five different concentrations per drug for 48 h and their viability was assessed by CellTiter-Glo assay, which quantifies metabolic activity (Fig. 1a, Supplementary Fig. 1a; see Supplementary Table 2 for raw data). Unbiased hierarchical clustering of both the cell lines and the compounds revealed that (1) compounds acting on the same pathway tend to cluster together, (2) only approximately one-third of the compounds reduces the viability of at least three cell lines, and (3) that cell lines of similar origin tend to cluster together (Fig. 1a, b, Supplementary Fig. 1a). We found that a small minority of drugs kill all cell lines; these tend to be chemotherapeutic agents such as doxorubicin, gemcitabine or docetaxel; in contrast, highly target-specific compounds differed strongly in terms of their cytotoxic activity (Fig. 1b). The differential susceptibility patterns correlated reasonably well with the cell-of-origin-based ABC-/GCB-DLBCL stratification that is currently used in clinical practice to assess the prognosis of a patient and to guide treatment decisions, with five of the six GCB-DLBCL cell lines clustering together, and away from the ABC-DLBCL cell lines (Fig. 1b). The only class of drugs showing cytotoxicity patterns that correlated with ABC-/GCB-DLBCL subtype was the AKT inhibitors ipatasertib and GSK690693, which selectively killed cell lines of the GCB-, but not the ABC-DLBCL subtype (Fig. 1b, c). The individual validation of select compounds largely confirmed the results of the screen (Fig. 1d).

To prove that on-target effects of the selected compounds were responsible for the differential killing of DLBCL cell lines, we performed competitive proliferation assays for which ten DLBCL cell lines were subjected to genome editing of the loci that encode the three above-mentioned selected drug targets, i.e., dihydrofolate reductase (DHFR), Bcl-2 and the AKT isoforms 1–3. Interestingly, only cell lines that were identified in the screen to be sensitive to AKT inhibition were, if subjected to *AKT2* deletion, outcompeted over a 24-day time course by their non-targeted, wild-type counterparts; the deletion of *AKT1* or *AKT3* had no effect (Supplementary Fig. 1b). The same differential competitive advantage of wild-type cells was observed upon *DHFR* deletion in methotrexate-sensitive, but not resistant cell lines (Supplementary Fig. 1b), and only the venetoclax-sensitive cell line RIVA showed evidence of a competitive advantage of wild-type over mutant cells (Fig. 1e, Supplementary Fig. 1c). In general, the results of the pharmacological and genetic approaches correlated very well as judged by their positive correlation (Supplementary Fig. 1d). The combined data suggest that drug response profiling is effective at exposing differential drug susceptibilities.

### Differential drug susceptibilities are attributable to underlying genetic aberrations and can be confirmed in vivo in humanized mouse models of DLBCL

We have previously subjected the DLBCL cell lines included in the drug screen described above to an in depth characterization of their mutational landscapes [17]. Interestingly, the selective sensitivity to AKT inhibition could be attributed to inactivating point mutations in *PTEN*, a tumor suppressor and negative regulator of the AKT signaling pathway, as the cell lines harboring a mutant *PTEN* allele [17] were among the most AKT inhibitor-sensitive investigated (Fig. 1d, Supplementary Fig. 2a). Interestingly, these same cell lines, in contrast to all others, expressed very little if any PTEN as determined by Western blotting (Supplementary Fig. 2a). In contrast, neither AKT expression nor its auto-phosphorylation on threonine T308 were particularly good predictors of AKT inhibitor sensitivity (Supplementary Fig. 2b).

**Fig. 2:**
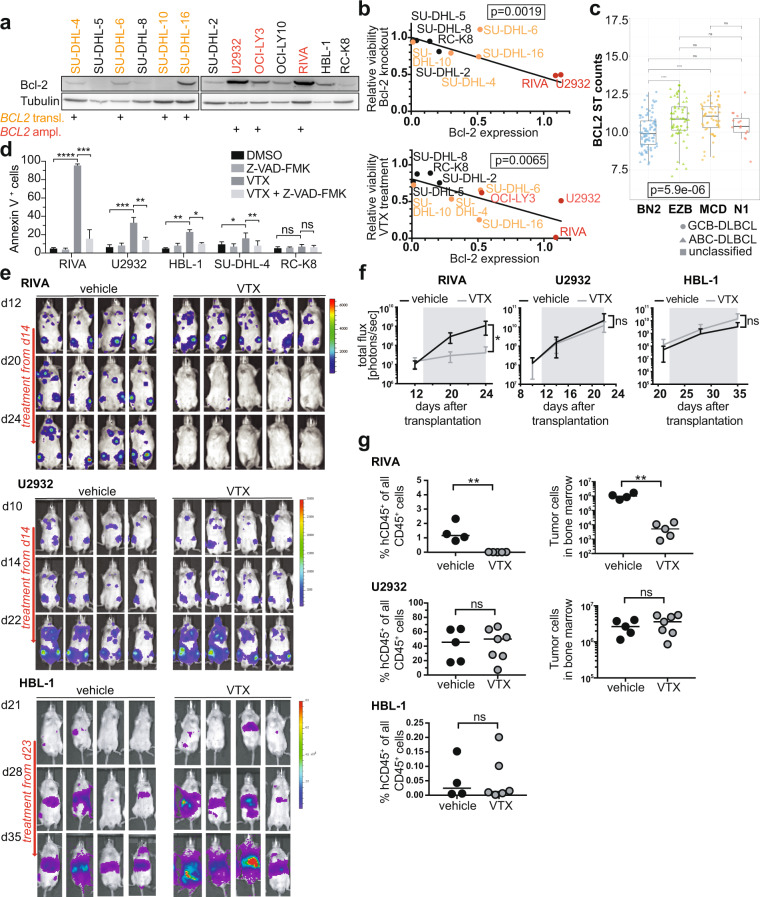
The level of Bcl-2 expression determines venetoclax sensitivity of DLBCL cell lines in vitro and in vivo. **a** Bcl-2 expression of the indicated cell lines as determined by Western blotting; tubulin expression served as loading control. The mutational status of the indicated genes is annotated below the western blots; the color code indicates the *BCL2* status (yellow: *BCL2* translocation; red: *BCL2* amplification; black: wild-type *BCL2*). **b** Inverse correlation of Bcl-2 expression with the viability after *BCL2* gene editing (upper panel) and venetoclax exposure (lower panel), of the indicated cell lines. The *p* values were determined by log-rank test. **c** *BCL2* gene expression of 206 DLBCL cases available through TCGA, stratified based on genetic subtype as assigned by Schmitz et al. [10]. The *p* value was determined by Kruskal–Wallis test, and pairwise *p* values by Mann–Whitney–Wilcoxon test. Symbols indicate the subtype based on gene expression signature. **d** Apoptosis rates as determined by annexin V staining, of the indicated cell lines exposed to 40 nM venetoclax and/or the pan-caspase inhibitor z-VAD-FMK. Data are presented as mean ± standard deviation of three independent experiments. *p < 0.05, **p < 0.01, ***p < 0.005, ****p < 0.001, as determined by one-way ANOVA. **e**–**g** MISTRG (RIVA, U2932) and MISTRG6 (HBL-1) mice were injected intravenously with 1 × 10^7^ cells of the three indicated cell lines; IVIS images were recorded on the indicated days (**e**) and the radiance was plotted longitudinally as means ± SD (**f**). Mice received twice-weekly doses of 40 mg/kg venetoclax via oral gavage, initiated once lymphomas were clearly detectable in all mice of the cohort (after two and three weeks of growth, respectively; indicated by gray shading in **f**). The lymphoma burden was quantified in the bone marrow at the study endpoint by flow cytometric staining for human (hCD45) and mouse CD45 (4–5 weeks post injection, in **g**). Data are representative of at least two independent experiments; only one study was conducted with HBL-1 cells. Horizontal lines indicate medians. **p < 0.01, as determined by Mann–Whitney test. ns, not significant.

Aberrant Bcl-2 expression in DLBCL can be caused by either the chromosomal t(14;18) translocation, which juxtaposes *BCL2* to *IgH* gene enhancer elements and affects ~20% of GCB-DLBCL but is rare in ABC-DLBCL, or by *BCL2* amplification in ABC-DLBCL [10]. Of six examined DLBCL cell lines of the ABC subtype, three harbor *BCL2* amplifications; these are the cell lines with the highest Bcl-2 expression (Fig. 2a). Of the six examined GCB-DLBCL cell lines, four harbor the t(14;18) translocation and express intermediate Bcl-2 protein levels (Fig. 2a). The ABC- and GCB-DLBCL cell lines with wild-type *BCL2* alleles express the lowest Bcl-2 protein levels (Fig. 2a). Bcl-2 expression correlated very well with both venetoclax susceptibility and viability upon genetic ablation as determined by competitive proliferation assay (Fig. 2b). Of 206 DLBCL cases whose transcriptomic and whole exome data are available through TCGA, samples of the «MCD» and «EZB» genetic subtype expressed the highest Bcl-2 transcript levels, and samples of the «BN2» subtype had the lowest Bcl-2 expression (Fig. 2c). Venetoclax reduced the viability of RIVA cells by inducing apoptosis as determined by annexin V staining; venetoclax-induced apoptosis coincides with the loss of mitochondrial membrane potential and can be prevented completely by the pan-caspase inhibitor z-VAD (Fig. 2d). The Bcl-2-negative cell lines RC-K8 and SU-DHL-4 did not undergo apoptosis upon exposure to venetoclax, and the Bcl-2^hi^ and Bcl-2^int^ cell lines U2932 and HBL-1 showed an intermediate phenotype (Fig. 2d).

We next asked whether the differential susceptibility to venetoclax can be recapitulated in vivo in a suitable xenotransplantation model of DLBCL. To this end, luciferase-expressing RIVA, U2932 and HBL-1 cells were injected into genetically humanized mice [12]. All three cell lines engrafted well in such MISTRG (U2932, RIVA) or MISTRG6 (HBL-1) mice, respectively, and their growth in spleens, bone marrow, and various non-lymphoid organs such as kidneys, reproductive tract, lung, and liver could readily be followed over time by in vivo imaging (IVIS, Fig. 2e). We initiated venetoclax treatment once tumors were clearly visibly by IVIS in all mice; interestingly, in mice transplanted with RIVA cells but not in mice transplanted with U2932 or HBL-1 cells, two to three doses of intragastrically administered venetoclax were sufficient to reduce the lymphoma burden to below the IVIS detection limit (Fig. 2e,f) and to significantly reduce the frequency of human lymphoma cells in bone marrow and spleen at the study endpoint (Fig. 2g). The combined results demonstrate that high Bcl-2 expression due to *BCL2* amplification and/or translocation is required, but not sufficient for responses to venetoclax in vitro and in vivo.

### BCL2 and MCL1 expression are mutually exclusive and druggable in DLBCL

**Fig. 3:**
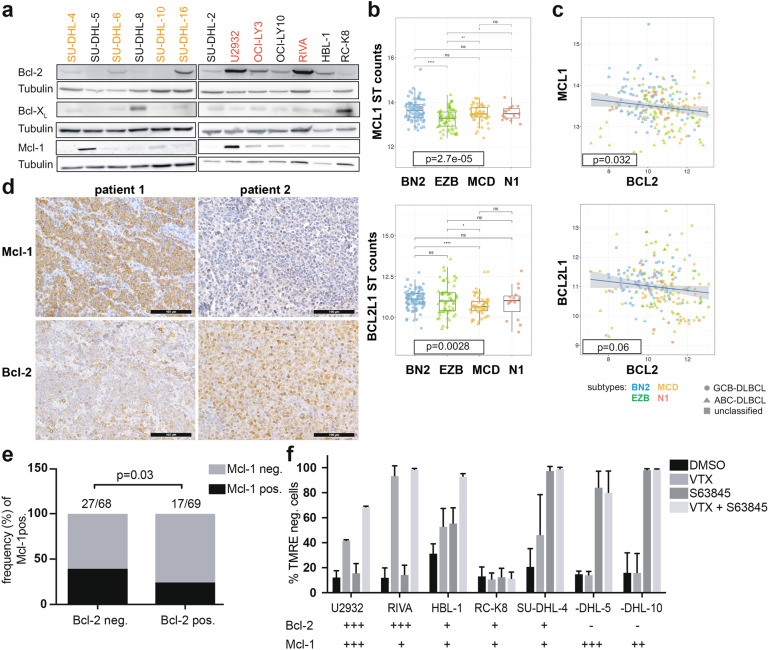
Bcl-2 and Mcl-1 are mutually exclusively expressed and functionally redundant in DLBCL. **a** Bcl-2, Bcl-X_L,_ and Mcl-1 expression of the indicated cell lines as determined by Western blotting; tubulin expression served as loading control. Note that the Bcl-2 western blot is the same as shown in Fig. 2a, and is included again here to facilitate the comparison. **b** *MCL1* and *BCL2L1* gene expression of 206 DLBCL cases available through TCGA, stratified based on genetic subtype as assigned by Schmitz et al. [10]. The global *p* value was determined by Kruskal–Wallis test, and pairwise p-values by Mann–Whitney-Wilcoxon test. **c** Inverse correlation of *MCL1* and *BCL2* expression, and of *BCL2L1* and *BCL2* expression, of the 206 cases shown in b. **d**, **e** Mcl-1 and Bcl-2 expression, as determined by immunohistochemistry of 137 DLBCL patients. Representative pairs of stainings are shown in d for two cases, of whom one (patient 1) was Mcl-1-positive and Bcl-2-negative, and one (patient 2) was Mcl-1-negative and Bcl-2-positive (scale bar, 100 μm). The fraction (in %) of Mcl-1-positive among all Bcl-2-negative and -positive cases is shown in **e**, with absolute numbers listed above the respective graphs. The cutoffs for assigning Bcl-2 and Mcl-1 positivity were >70% and >30% of tumor cells, respectively. The inverse correlation (correlation coefficient: −0.161) is statistically significant as determined by Spearman correlation. **f** Viability as assessed by TMRE staining for mitochondrial membrane potential of the indicated cell lines, after 48 h of exposure to 40 nM venetoclax and/or to 1 μM S63845, an Mcl-1 inhibitor. The Mcl-1 and Bcl-2 expression of each cell line is annotated below the respective graphs on a scale from − to +++. Data in **f** are pooled from two and up to three independent experiments and plotted as means ± standard deviation. TMRE labels live cells with intact mitochondrial membrane potential; TMRE-negative cells are considered apoptotic.

We speculated that the resistance of DLBCL cells to venetoclax might be due to the expression of other anti-apoptotic members of the Bcl-2 family of proteins. Indeed, U2932 and another DLBCL cell line, SU-DHL-5, cells were found to express high levels of Mcl-1 as determined by Western blotting, and two other cell lines (SU-DHL-8 and RC-K8) expressed very high levels of Bcl-X_L_ (Fig. 3a). Mcl-1 and Bcl-X_L_ (encoded by the *BCL2L1* gene) transcript levels were highest in the «BN2» genetic subtype (Fig. 3b), which had shown the lowest Bcl-2 transcript levels (Fig. 2c), indicating that the genetic subtypes of DLBCL rely on different anti-apoptotic Bcl-2 family members. Mcl-1 expression, and to a lesser extent Bcl-X_L_ expression, was inversely correlated with Bcl-2 expression at the transcriptional level in these 206 DLBCL patients (Fig. 3c). An inverse association, and mutually exclusive expression of Bcl-2 and Mcl-1 was confirmed in a second cohort, and at the protein level, by IHC staining of a tissue microarray comprising 137 DLBCL samples for Mcl-1 and Bcl-2 (Fig. 3d, e). Interestingly, the Mcl-1 inhibitor S63845, a selective BH3-mimetic [18], was effective at killing DLBCL cells with no or low expression of Bcl-2 only, and synergized with venetoclax in killing a DLBCL cell line with co-expression of both Bcl-2 family proteins (Fig. 3f). In summary, these results suggest that DLBCL cells rely on Bcl-2, Mcl-1 or Bcl-X_L_ for resistance to apoptosis, and these vulnerabilities can be readily identified and exploited for treatment purposes.

### BTK inhibition sensitizes Bcl-2-expressing DLBCL cells to venetoclax and overcomes primary resistance in vitro and in vivo

**Fig. 4:**
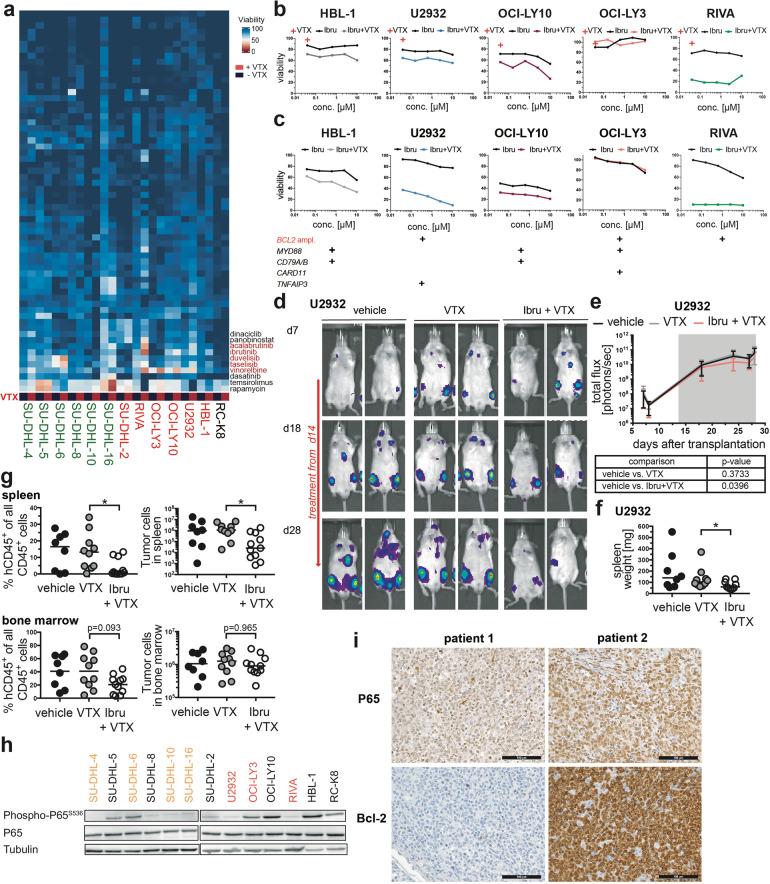
BTK inhibition synergizes with Bcl-2 inhibition to kill DLBCL cells with constitutively active BCR signaling in vitro and in vivo. **a** Heat map displaying the viability (calculated as the mean of the five concentrations assessed per drug) of 13 DLBCL cell lines, as determined by CellTiter-Glo viability assay, after 48 h of exposure to 62 manually selected compounds, with or without additional exposure to 40 nM venetoclax. Select compounds that synergize with venetoclax to kill DLBCL cells are annotated. Five concentrations, generated by serial (fourfold) dilution, were tested per compound. See Supplementary Table 3 for the compounds and their respective highest concentrations. **b** Viability curves, derived from the drug screen, of the indicated cell lines, showing all five assessed concentrations of ibrutinib, either alone or in combination with venetoclax. The red cross indicates the viability upon exposure to 40 nM venetoclax as single agent. **c** Validation of viability after 48 h of exposure to ibrutinib alone or in combination with venetoclax, as assessed individually for each cell line by CellTiter-Blue assay. A representative experiment of two independent ones per cell line is shown as mean viability of duplicate measurements. The mutational status of the indicated genes is shown below the graphs for each cell line. **d**–**g** MISTRG6 mice were injected intravenously with 1 × 10^7^ U2932 cells; IVIS images were recorded on the indicated days (d) and the radiance was plotted longitudinally as means ± SD (**e**). Mice received twice-weekly doses of 40 mg/kg venetoclax, either alone or in combination with 25 mg/kg ibrutinib via oral gavage, initiated once lymphomas were clearly detectable in all mice of the cohort (after two weeks of growth; indicated by gray shading in **e**). Spleen weights were recorded (**f**) and the lymphoma burden was quantified in the spleen (upper panel) and bone marrow (lower panel) at the study endpoint by flow cytometric staining for human (hCD45) and mouse CD45 (4 weeks post injection, in **g**). Data are pooled from two independent experiments. *p < 0.05, as determined by Mann–Whitney test. **h** P65 expression and phosphorylation in the indicated cell lines, as determined by Western blotting; tubulin expression served as loading control. **i** Bcl-2 and nuclear P65 expression, as determined by immunohistochemistry of 39 DLBCL patients. Representative pairs of stainings are shown for two cases, of whom one (patient 1) is P65-negative and Bcl-2-negative, and one (patient 2) is moderately positive for nuclear P65 and is Bcl-2-positive (scale bar, 100 μm).

We next set out to systematically search for compounds that would synergize with venetoclax to overcome primary resistance. To this end, we conducted a combinatorial screen for which all 13 DLBCL cell lines were exposed to 62 manually selected, FDA-approved compounds in five different concentrations (Supplementary Table 3), which were either administered alone or in combination with venetoclax. The vast majority of compounds showed no differential cytotoxicity when administered as single agents relative to their combination with venetoclax (Fig. 4a, Supplementary Fig. 3a; see Supplementary Table 4 for raw data). However, the Bruton’s tyrosine kinase (BTK) inhibitors ibrutinib and acalabrutinib sensitized a subset of DLBCL cell lines to venetoclax across all five examined concentrations (Fig. 4a, b, Supplementary Fig. 3a, b). We identified three distinct types of responses to venetoclax/(acala)ibrutinib single and combination therapy: (1) cell lines with wild-type *BCL2* alleles and no or minimal Bcl-2 expression were totally resistant to venetoclax, also in the combination with ibrutinib (SU-DHL-2, −5, −8, RC-K8); (2) cell lines with moderate Bcl-2 expression due to the t(14;18) translocation were primarily resistant to venetoclax, but could be sensitized by ibrutinib (SU-DHL-4, −16; exception SU-DHL-10); (3) cell lines with high Bcl-2 expression either responded (RIVA) or not (U2932, OCI-LY10, HBL-1) to single-agent venetoclax, but were all readily killed by the combination treatment (Fig. 4a, b, Supplementary Fig. 3a, b). The screening data could be validated in individual viability experiments (Fig. 4c). Interestingly, both ABC- and GCB-DLBCL cell lines responded to the combined treatment. As comparatively high concentrations of ibrutinib were used in our drug screen and its validation, we additionally exposed four cell lines to the venetoclax/ibrutinib combination at concentrations that were more in line with the reported IC50 of ibrutinib (for BTK, as determined by in vitro biochemical kinase assays), which ranges from 0.18 to 1.5 nm [19]. Indeed, we found ibrutinib to sensitize cell lines such as HBL-1 and U2932 to venetoclax at concentrations as low as 2.5 and even 0.65 nm (Supplementary Fig. 3c–f). The two drugs showed at least additive (synergy score between 0 and 10), and for some concentrations synergistic (score > 10) effects (Supplementary Fig. 3c–f), as determined by two complementary, commonly used methods to define synergy (HAS, BLISS) [20].

The only two additional compound classes found in our screen to synergize with venetoclax in killing DLBCL cell lines were the PI3K inhibitors duvelisib and taselisib, as well as the mitosis inhibitor vinorelbine (Fig. 4a, Supplementary Fig. 3a). We were able to confirm the synergy of PI3K inhibition with venetoclax using two PI3K inhibitors that are in advanced clinical development, duvelisib (that had also been included in the drug screen) and idelalisib (Supplementary Fig. 3g, h). Cell lines with moderate to high Bcl-2 expression all responded to venetoclax in combination with either idelalisib or duvelisib (with one exception: OCI-LY3), whereas cell lines with no evidence of Bcl-2 expression did not (Supplementary Fig. 3g, h).

To assess whether venetoclax synergizes with ibrutinib also in vivo, we injected MISTRG6 mice with U2932 cells and initiated single agent and double agent treatments once lymphomas were clearly detectable by IVIS. U2932-derived tumors progressed more slowly on combination treatment as judged by IVIS (Fig. 4d, e) and by their spleen weights and the flow cytometric quantification of human lymphoma cells in the spleen and bone marrow at the study endpoint (Fig. 4f, g); ibrutinib alone had no effect on the tumor burden (Supplementary Fig. 3i, j). Similar results were obtained in RIVA cells (see next chapter). The constitutive activation of canonical NF-κB signaling turned out to be an accurate predictor of treatment response, as several cell lines responsive to the venetoclax/ibrutinib combination showed evidence of phosphorylated P65 (Fig. 4h). Among a cohort of 39 DLBCL patients for whom both Bcl-2 expression and nuclear P65 localization were evaluable by immunohistochemistry, 18 (46%) had strong Bcl-2 expression in more than 70% of tumor B-cells (Fig. 4i); of these, 6 (33%) had either weakly (4) or moderately (2) positive nuclear P65 signals (Fig. 4i), and would therefore be predicted to benefit from combined Bcl-2/BTK inhibition. We also examined a possible synergy in vivo of venetoclax and the PI3K inhibitor duvelisib: indeed, only the combination, but not venetoclax alone, reduced the tumor burden in the bone marrow and in other affected organs in mice xenotransplanted with SU-DHL-16 cells (Supplementary Fig. 3k, l). Our results thus provide a strong rationale for combined Bcl-2 and BTK or PI3K inhibition in subgroups of patients that harbor *BCL2* amplifications or translocations and therefore express high levels of Bcl-2, and that additionally have evidence of constitutively active NF-κB activation.

### Ibrutinib reduces the expression of various anti-apoptotic genes in susceptible, but not resistant DLBCL cell lines in vitro and in vivo

**Fig. 5:**
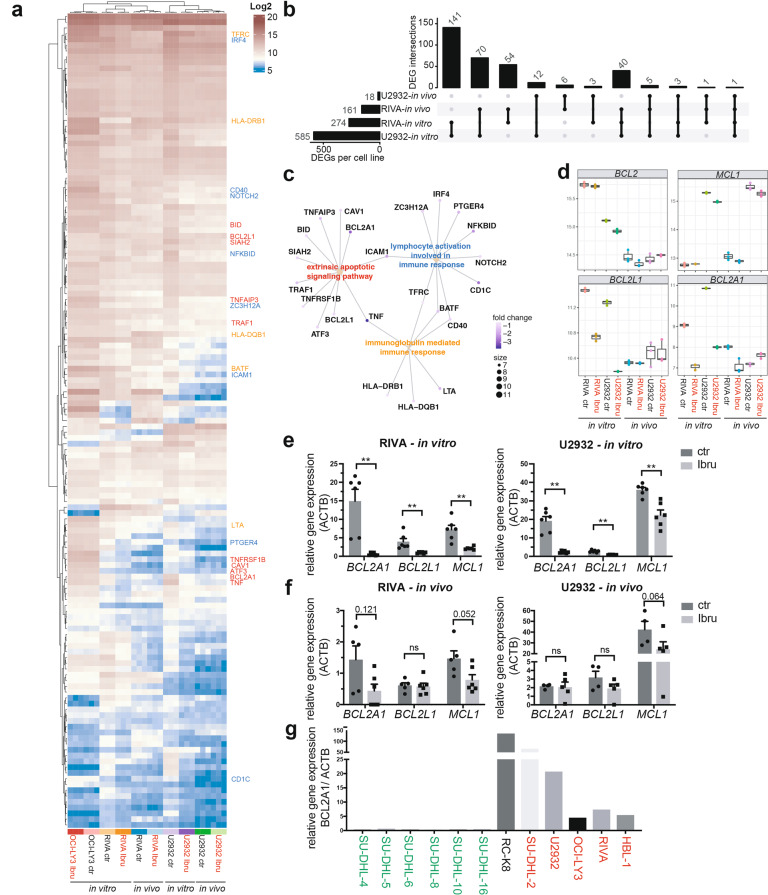
Transcriptional profiling reveals a BTK-dependent signature of anti-apoptotic gene expression. **a**–**d** RIVA, U2932 and OCI-LY3 cells were cultured in the presence or absence of ibrutinib for 6 h and subjected to RNA isolation; U2932 and RIVA cells were additionally orthotopically transplanted into six MISTRG mice per cell line and subjected to two weeks of continuous in vivo ibrutinib exposure (25 mg/kg, administered five times per week). Bone marrow cells were harvested from three ibrutinib-treated and three vehicle-treated control mice, and tumor cells were FACS-sorted to >95% purity prior to RNA isolation. In vitro and in vivo generated triplicate samples were subjected to bulk RNA sequencing. A heat map of 141 genes that are downregulated (log2 fold change <−0.5 and adjusted *p* value < 0.05) upon ibrutinib exposure in vitro in both RIVA and U2932 cells are shown for all 30 samples in **a**. Select genes are annotated (color code as in **c**). An UpSet plot of the intersections of overlapping differentially expressed, downregulated genes across the four indicated conditions is shown in **b**. The top bar chart indicates the number of downregulated genes shared between two, three, or all four conditions. Three select gene networks as identified by pathway analysis of the 141 commonly downregulated genes are shown in **c**, connecting the indicated genes assigned to each pathway (color-coded in yellow, blue and red as in **a**). The color of each dot indicates the average fold change in expression of the respective gene; its size indicates the number of genes from the list of 141 that are in the respective pathway. Whisker plots of the variance stabilized transformed expression of four select anti-apoptotic Bcl-2 family members (*BCL2, MCL1, BCL2L1,* and *BCL2A1*) are shown in **d** for all triplicate samples of eight conditions. **e**, **f** quantitative RT-PCR of the three indicated transcripts, as determined using an independently generated set of in vitro (6 h of exposure) and in vivo ibrutinib-exposed RIVA and U2932 cells. Each dot represents one sample; bars represent means + standard deviation. P-values were determined by Mann–Whitney test. **g** *BCL2A1* gene expression of the indicated cell lines as determined by qRT-PCR; ABC-DLBCL cell lines are color-coded in red, and GCB-DLBCL cell lines in green; an unclassified cell line, RC-K8, is shown in black.

In order to systematically assess the mechanistic basis of the venetoclax/ibrutinib synergy, we subjected two susceptible (i.e. to the combination; U2932 and RIVA) and one resistant (OCI-LY3) cell line to ibrutinib exposure and performed bulk RNA sequencing after 6 h of exposure in vitro or two weeks of continuous treatment in vivo. Principle component analysis (Supplementary Fig. 4a) and unsupervised hierarchical clustering based on the top 2000 differentially expressed genes (DEGs, Supplementary Fig. 4b) revealed that cell line identity is the main driver of sample segregation, followed by in vitro vs. in vivo growth and exposure to ibrutinib vs. vehicle. The three replicate samples per condition were virtually identical and clustered together tightly without exception (Fig. 5a, Supplementary Fig. 4b, c). The vast majority of DEGs was down- as opposed to upregulated. Whereas only 54 genes were differentially expressed and downregulated (i.e. with a log2 fold change <−0.5 and adjusted *p* value < 0.05) in the resistant OCI-LY3 cell line under in vitro exposure to ibrutinib (data not shown), this number was much higher for the sensitive cell lines (585 for U2932, 274 for RIVA; Fig. 5b). A substantial overlap of 141 commonly downregulated genes was detected between the two sensitive cell lines, of which 40 genes could be confirmed in at least one cell line also in vivo (Fig. 5a, b). The 141 differentially downregulated genes could be assigned by network analysis to numerous significantly enriched pathways, such as extrinsic apoptotic signaling, lymphocyte activation and immunoglobulin-mediated immune response (Fig. 5c). Several NF-κB target genes were identified among the differentially downregulated genes (*TNFAIP3*, *TNF*, *ICAM1*, *CD40* and others). Whereas the expression of *BCL2* and *MCL1* was not found to be controlled by ibrutinib in this unbiased analysis, neither in vitro nor in vivo, two other anti-apoptotic Bcl-2 family members, *BCL2L1* (encoding Bcl-X_L_) and *BCL2A1* (encoding Bcl-2 related protein A1) were strongly downregulated in at least three of the four examined conditions (Fig. 5d). The differential expression of *BCL2L1* and *BCL2A1* could be confirmed by quantitative RT-PCR with an independently generated set of in vitro and/or in vivo ibrutinib-exposed samples (Fig. 5e, f). Interestingly, the expression of *BCL2A1* was strictly limited to the ABC-DLBCL subtype in our cell line panel (Fig. 5g), which is more likely to harbor mutations driving BTK-dependent chronic active BCR signaling. In summary, unbiased RNA-sequencing-based transcriptome analyses reveal possible clues to the mechanistic basis of the ibrutinib/venetoclax synergy, and specifically point to the anti-apoptotic Bcl-2 family members Bcl-X_L_ and Bcl-2A1 as drivers of alternative survival pathways that must be overcome for efficient killing of Bcl-2-expressing DLBCL cells by venetoclax.

### Venetoclax/ibrutinib combination therapy salvages mice with recurring lymphoma after single agent venetoclax treatment

**Fig. 6:**
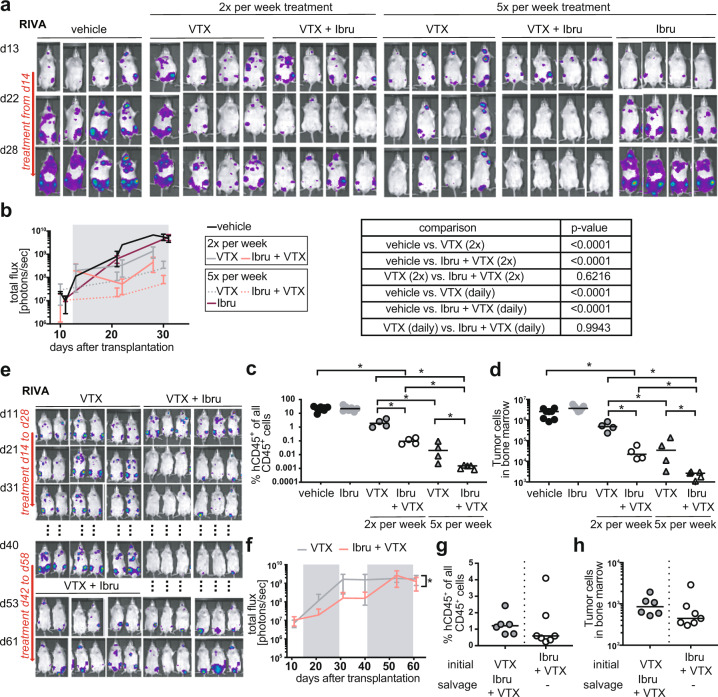
Combined Bcl-2 and BTK inhibition delays and overcomes secondary venetoclax resistance in vivo. **a**–**d** MISTRG mice were injected intravenously with 1 × 10^7^ RIVA cells; IVIS images were recorded on the indicated days (**a**) and the radiance was plotted longitudinally over time (**b**). IVIS images are shown for four representative mice per condition in **a**, and means ± SD are shown for all four to seven mice per treatment condition in **b**, along with the *p* values for the relevant comparisons (determined by one-way ANOVA of AUC). Mice received either two or five doses per week of 40 mg/kg venetoclax, either alone or in combination with 25 mg/kg ibrutinib via oral gavage, initiated once lymphomas were clearly detectable in all mice of the cohort (after two weeks of growth; treatment window indicated by gray shading in **b**). All mice were subjected to the quantification of their lymphoma burden in the hind leg bone marrow at the study endpoint by flow cytometric staining for hCD45 and mouse CD45. Human tumor cell frequencies among all (human and mouse) CD45^+^ cells are shown in **c**, and absolute counts are shown in **d**. *p < 0.05, **p < 0.01, ***p < 0.005, as determined by one-way ANOVA. **e**–**h** MISTRG mice were injected intravenously with 1 × 10^7^ RIVA cells, and were treated as described in **a**–**d** with five doses per week of venetoclax or venetoclax plus ibrutinib. After an interval of two weeks without drug treatment leading to lymphoma progression in a subset of mice, combination salvage therapy was re-initiated for another two weeks where indicated. IVIS images are shown for all six mice per condition in **e**, and means ± SD are shown in **f**. The flow cytometric quantification of the lymphoma burden in the hind leg bone marrow is shown in **g** and **h** for the study endpoint at 9 weeks post injection.

We next set out to collect more detailed information on the optimal in vivo dosing of venetoclax and ibrutinib and their combination, and the long-term effects of continuous vs. interrupted treatment on the development of secondary resistance. The combination treatment reduced the tumor burden more efficiently than venetoclax as single agent, irrespective of whether the compounds were administered daily or twice-weekly (Fig. 6a–d), recapitulating our observations in U2932 cells (Fig. 4d–g). Daily treatment was clearly more effective than twice-weekly treatment (Fig. 6a–d), and ibrutinib -even when administered daily- had no effect whatsoever on its own (Fig. 6a–d, Supplementary Fig. 5a). Venetoclax/ibrutinib synergy was observed in three independent studies using RIVA cells (Fig. 6a–f, Supplementary Fig. 5b–d). We next asked how durable the response to venetoclax or its combination with ibrutinib would be if the treatment were discontinued upon tumor cell eradication. Mice were treated with venetoclax or venetoclax/ibrutinib until the IVIS-detectable tumor burden was at or close to zero in mice on the combination, at which time the treatment was discontinued. Regular IVIS imaging thereafter revealed tumor recurrence in all (11/11) mice that had been on venetoclax alone, and even -albeit delayed- in all mice on the combination (11/11), at three weeks into the treatment break (Fig. 6e, f, Supplementary Fig. 5b, c). The re-initiation of venetoclax treatment alone after three weeks of treatment break stabilized, but failed to eradicate the lymphoma burden (Supplementary Fig. 5b, c); the re-initiation of venetoclax/ibrutinib combination treatment effectively salvaged mice with recurring lymphoma (Fig. 6e–h) and prevented or strongly reduced recurrence after the treatment-free interval (Supplementary Fig. 5b–d). The combined results lead us to conclude that (1) daily treatment is superior to twice-weekly treatment, and well tolerated; (2) the combination therapy of venetoclax with ibrutinib is superior to the single agents in eliminating the primary tumor, in preventing or delaying recurrence, and in the salvage therapy setting; and (3) that any therapy, including the combination, is ideally administered in a continuous, uninterrupted fashion.

### Primary DLBCL cells respond to the ibrutinib/venetoclax combination in vivo

**Fig. 7:**
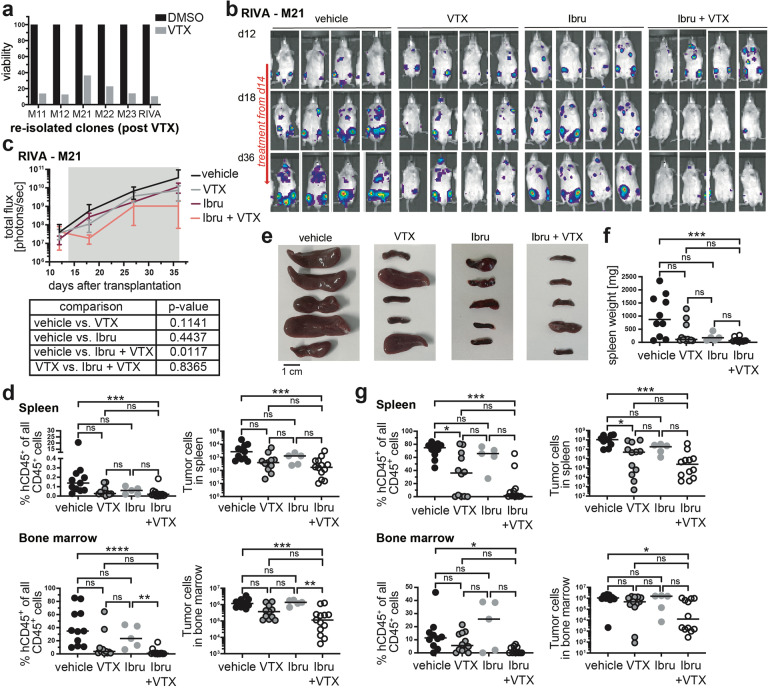
Acquired venetoclax resistance can be overcome by ibrutinib addition in vivo. **a** RIVA cells immunomagnetically isolated from five different donors (that had been exposed during weeks three and four, and again during weeks seven and eight to venetoclax as single agent) were exposed to 40 nM venetoclax for 48 h and subjected to viability testing by CellTiter-Blue assay; the original cell line is shown as control. **b**–**d** Venetoclax-resistant RIVA cells isolated from donor mouse M21 were re-transplanted into MISTRG recipients. IVIS images were recorded on the indicated days (**b**) and the radiance was plotted longitudinally as means ± SD (**c**); *p* values for the relevant comparisons (as determined by one-way ANOVA of AUC) are shown below the curves. Mice received twice-weekly doses of 40 mg/kg venetoclax, 25 mg/kg ibrutinib, or the combination via oral gavage, initiated once lymphomas were clearly detectable in all mice of the cohort (after 2 weeks of growth; indicated by gray shading in **c**). All mice were subjected to the quantification of their lymphoma burden in the spleen and bone marrow at the study endpoint by flow cytometric staining for hCD45 and mouse CD45 (**d**). Both the frequencies and absolute counts are shown. Data in c and d are pooled from three independent experiments (the ibrutinib-only group was included only once); representative images from one study are shown in (**b**). **e**–**g** 1 × 10^6^ human primary DLBCL cells were transplanted into MISTRG6 mice, which were treated from week four post transplantation onwards with either venetoclax, ibrutinib or the combination as described for b-d. Images of the spleens, and their weights are shown in **e** and **f**; the human lymphoma burden in the spleen and bone marrow at the study endpoint (eight weeks post transplantation) is shown in **g**. Data in **e**–**g** are pooled from two independent studies. *p < 0.05, **p < 0.01, ***p < 0.005, as determined by one-way ANOVA throughout.

Resistance to venetoclax has been linked to Bcl-X_L_ overexpression on the one hand [21] and mutations affecting the venetoclax binding site of Bcl-2 (G101V, Phe104Ile) on the other [22, 23]. Ex vivo resistance testing of lymphoma cells harvested from venetoclax-only-treated mice (Supplementary Fig. 5b–d) revealed varying degrees of resistance to venetoclax upon ex vivo exposure to venetoclax (Fig. 7a). Resistant cells harvested from five different donors expressed similar levels of Bcl-2 as the input RIVA cell line and showed no evidence of increased Mcl-1 or Bcl-X_L_ expression (Supplementary Fig. 6a, and data not shown). We could also not detect mutations in *BCL2* gDNA the output clones that affected the venetoclax binding site (Supplementary Fig. 6b). We used a comparatively venetoclax-resistant output population (from mouse 21) to address whether combining venetoclax with ibrutinib would overcome acquired resistance in vivo. This was indeed the case, as this resistant output population, upon re-transplantation, was successfully treated by the combination of venetoclax with ibrutinib, but continued to respond poorly to venetoclax or ibrutinib alone (Fig. 7b–d). The combined results suggest that our orthotopic xenotransplantation model lends itself to studies of venetoclax resistance, and show that ibrutinib effectively overcomes secondary, acquired resistance to Bcl-2 inhibition. Finally, we asked whether primary DLBCL cells from a patient with stage IVBE ABC-DLBCL with extranodal manifestations in the liver, an IPI score of 5 and a diagnosis of splenomegaly, which we had shown earlier to engraft readily in MISTRG6 mice [12] would respond to venetoclax or ibrutinib mono- or combination therapy. The combination was more effective than the mono-therapies at reducing the tumor burden or entirely eliminating the tumor cells from the spleen and bone marrow as judged by the spleen size and weight at the study endpoint (Fig. 7e, f) and the human tumor cell burden in the spleen and bone marrow (Fig. 7g). The combined data suggest that the venetoclax/ibrutinib combination is effective not only in orthotopic cell line transplantation models, but also in this patient-derived xenograft (PDX) setting.

## Discussion

Our data from single and combinatorial drug screens show that inhibitors of Bcl-2 and BTK or PI3K act synergistically to kill DLBCL cells that exhibit high Bcl-2 expression due to either *BCL2* translocations or amplifications. Aberrantly high Bcl-2 expression is a common hallmark of DLBCL, predominantly affecting cases and genetic subtypes featuring (1) co-occurring *MYD88*/*CD79* mutations and ABC-DLBCL origin, in which Bcl-2 overexpression is driven by gene amplification, or alternatively, featuring (2) *BCL2* translocations, co-occurring activating *EZH* mutations and a GCB-DLBCL cellular origin. Examples of both DLBCL subtypes were represented among our panel of DLBCL cell lines and could be used to confirm the genetic dependence of these - but not of Bcl-2^low^ - cell lines on Bcl-2 expression in competition experiments, for which the *BCL2* locus was selectively ablated by genome editing. The same cell lines, equipped with luciferase to allow for their tracking by IVIS, responded to venetoclax exposure in vitro and in vivo, albeit in most instances only in combination with ibrutinib or the PI3K inhibitor duvelisib. Bcl-2^low^ cell lines and primary tumors from DLBCL patients tend to alternatively express Mcl-1, which is inversely correlated with Bcl-2 at the transcript level; the DLBCL subtype featuring *NOTCH2* mutations and *BCL6* translocations (termed “BN2” [10]) is particularly prone to high Mcl-1 (and low Bcl-2) expression. An inverse association between Mcl-1 and Bcl-2 was further confirmed in a second DLBCL cohort by immunohistochemistry of 137 cases spotted on a tissue microarray. We found the Mcl-1 inhibitor S63845 [18] to selectively induce apoptosis in Bcl-2-negative or Bcl-2^low^ cell lines, but to be ineffective by itself in Bcl-2^hi^ cell lines, suggesting that Bcl-2 and Mcl-1 are mutually exclusively expressed and can substitute for one another in preventing apoptosis in DLBCL.

To systematically search for additional resistance mechanisms, we conducted a combinatorial screen in which 62 FDA-approved compounds were assessed for their ability to sensitize DLBCL cells to venetoclax-induced toxicity. Two categories of drugs emerged from this screen, which target BTK on the one hand (ibrutinib, acalabrutinib), and the phosphatidylinositol 3-kinase (PI3K; duvelisib, taselisib) on the other. We focused our in vivo studies mostly on the venetoclax/ibrutinib combination as ibrutinib is safe [24], but unfortunately not efficacious when added to R-CHOP in DLBCL patients [25]; clinical testing of the venetoclax/ibrutinib combination has been initiated with a first small phase I trial [26] and more data from relevant in vivo models is urgently needed to inform the design of upcoming larger trials. The combination not only efficiently killed many of our Bcl-2^int^ and Bcl-2^hi^ cell lines in vitro, but also led to faster and more complete tumor regression in vivo of cell lines with primary as well as acquired resistance to venetoclax. All available in vivo data thus lend support to the addition of ibrutinib to the venetoclax regimen in relapsed or refractory patients with the intention of overcoming primary resistance and delaying, overcoming or preventing acquired resistance to venetoclax. Simple immunohistochemical analysis of Bcl-2 and of phosphorylated or nuclear P65 as a marker of constitutive NF-κB activation are potentially useful indicators of efficacy of the combinatorial treatment; six of 39 patients in our DLBCL patient cohort that were evaluable for both markers had evidence of both high Bcl-2 expression and of nuclear distribution of NF-κB P65.

Preclinical evidence for the synergy between venetoclax and ibrutinib has been reported previously in studies that attempted to overcome ibrutinib resistance in follicular lymphoma and DLBCL [27], and other B-cell malignancies; [28, 29] ibrutinib was further shown to interact favorably with Bcl-2 family inhibitors as well as inhibitors of the PI3K-AKT-mammalian target of rapamycin signaling cascade, other B-cell receptor pathway inhibitors, and several components of chemotherapy in a high-throughput small-molecule combination screen designed to define potential therapeutic combinations for the ABC subtype of DLBCL [30]. A small clinical trial that aimed to investigate the efficacy and safety of ibrutinib and venetoclax in 13 relapsed/refractory (R/R) DLBCL patients with non-GCB subtype and Bcl-2 overexpression recently reported ORRs at two cycles of 61.5%, with 3 (23.1%) patients achieving complete remission (CR) and 5 (38.4%) patients achieving partial remission (PR) [26].

The mechanism of ibrutinib/venetoclax synergy remains a matter of debate. We found no evidence of differential regulation of Bcl-2 or Mcl-1 under ibrutinib, as shown previously by others [27]. Rather, bulk RNA sequencing conducted on DLBCL cells retrieved from mice under ibrutinib, or exposed in vitro to ibrutinib, points to Bcl-X_L_ and Bcl-2A1 as alternative Bcl-2 family members that can substitute for Bcl-2 unless transcriptionally downregulated by BTK inhibition. Our results are in line with the conclusion that two independent survival pathways, one acting at the mitochondrial membrane and preventing the pro-apoptotic activities of Bcl family proteins such as Bak, Bax and Bid, and the other acting at endolysosomal membranes relaying survival signals via the multiprotein supercomplex formed by MYD88, TLR9 and the BCR [31], have to both be disabled to efficiently kill DLBCL cells with adaptations targeting both pathways.

## Supplementary information

suppl. table 1

suppl. table 2

suppl. table 3

suppl. table 4

Supplemental figures and methods
