# Supplementary material for: Inhibitors of Bcl-2 and Bruton’s tyrosine kinase synergize to abrogate diffuse large B-cell lymphoma growth in vitro and in orthotopic xenotransplantation models
Source: Leukemia. 2021 Nov 18;36(4):1035–47. doi: 10.1038/s41375-021-01470-4 (PMC8979814; doi:10.1038/s41375-021-01470-4)
Supplement: Supplementary file 5 — Supplemental figures and methods [file 41375_2021_1470_MOESM5_ESM.docx]

**Suppl. Figure 1**


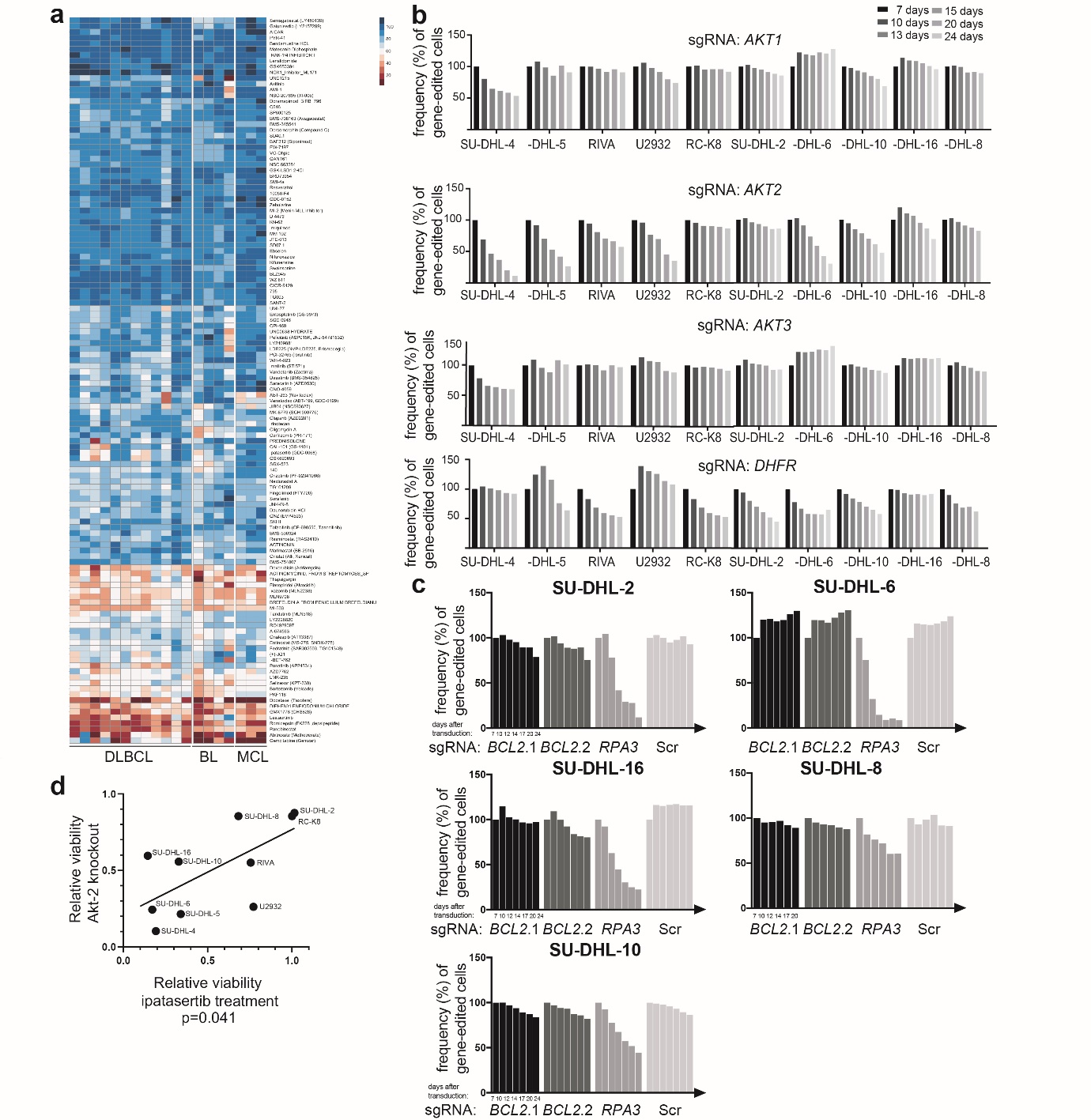


**Suppl. Figure 1. Competitive proliferation assays confirm the essential role of AKT and DHFR in DLBCL growth exposed by drug response profiling. a** Heat map displaying the viability (calculated as the mean of the five concentrations assessed per drug) of 19 cell lines of the indicated entities, as assessed by CellTiter-Glo viability assay, after 48 hours of exposure to 126 manually selected compounds targeting deregulated pathways in hematological malignancies. Five concentrations, generated by serial (five-fold) dilution, were tested per compound. MCL, mantle cell lymphoma; BL, Burkitt lymphoma. **b,c** Competitive proliferation assays, performed over a period of 24 days, of the indicated cell lines subjected to CRISPR-based editing of the *AKT1, AKT2, AKT3, DHFR* and *BCL2* loci, with *RPA* and a scrambled guide (Scr) serving as positive and negative controls. Successfully targeted cells were identified by their blue fluorescent protein (BFP) expression and mixed 1:1 with non-edited cells at the start of the experiment. Data are representative of three replicate experiments. **d** Correlation plots showing a positive correlation between the viability upon genetic loss of the gene of interest (*AKT2*) and the viability upon pharmacological targeting of the corresponding protein AKT-2 by ipatasertib.

**Suppl. Figure 2**

**
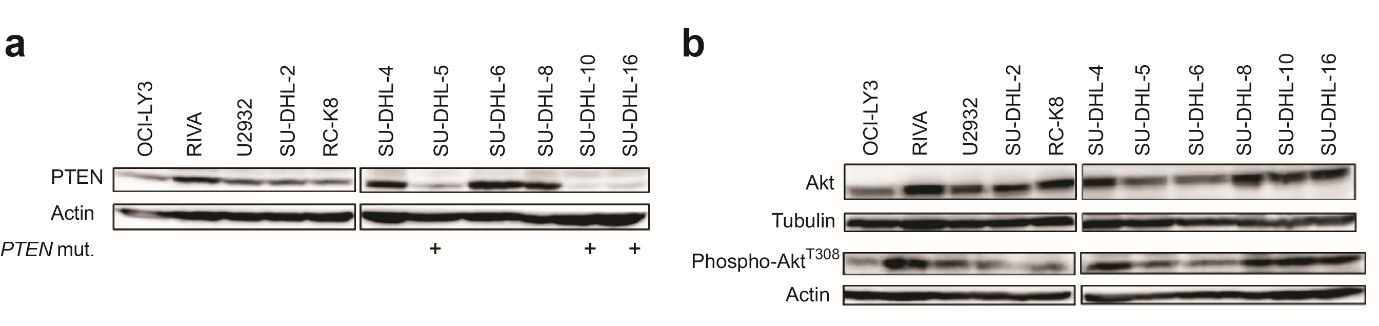
**

**Suppl. Figure 2. Differential drug responses to AKT inhibition can be attributed to differential PTEN but not AKT expression. a,b** PTEN and AKT expression and -phosphorylation of the indicated cell lines as determined by Western blotting; tubulin expression served as loading control.

**Suppl. Figure 3**

**
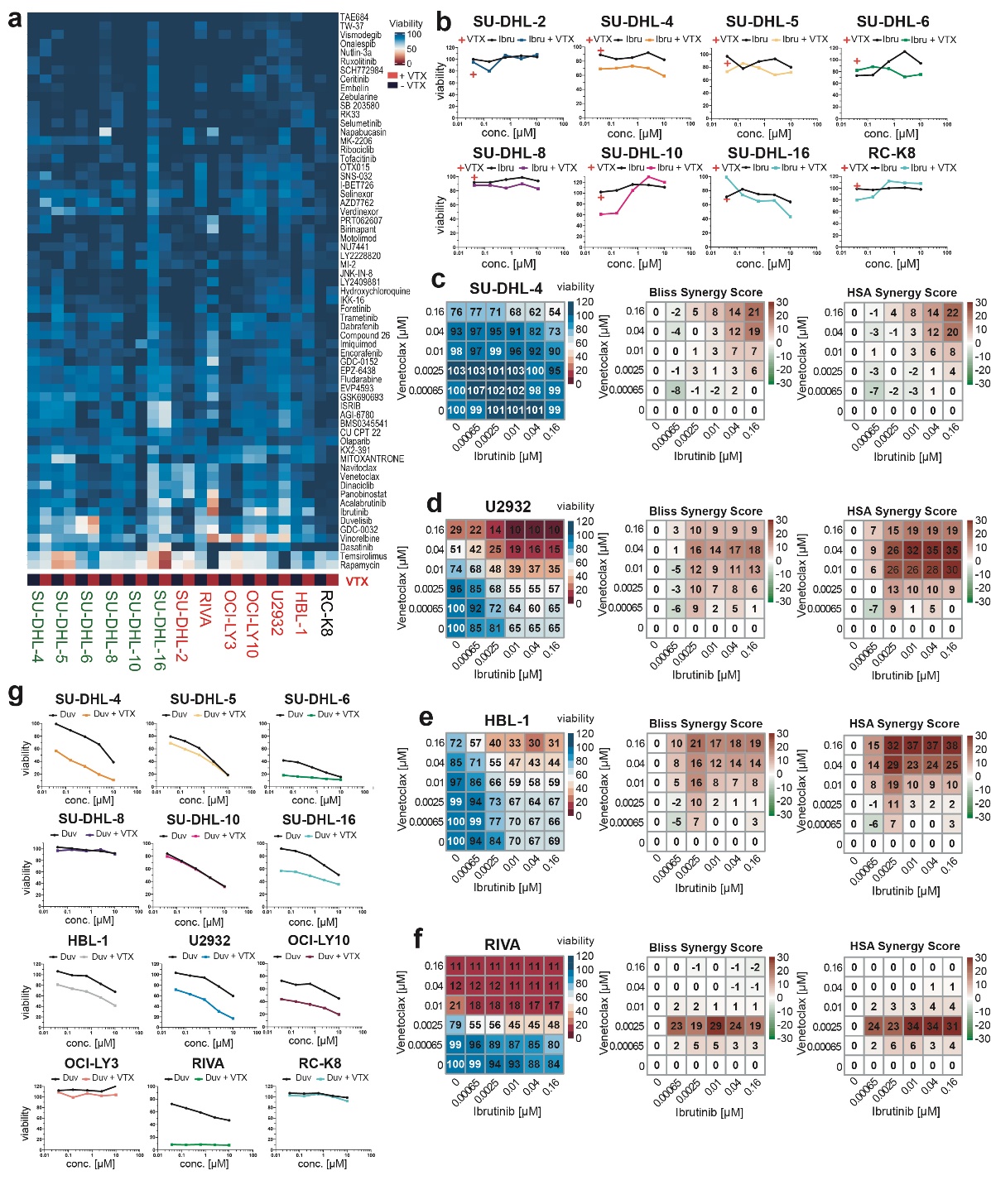
**

**
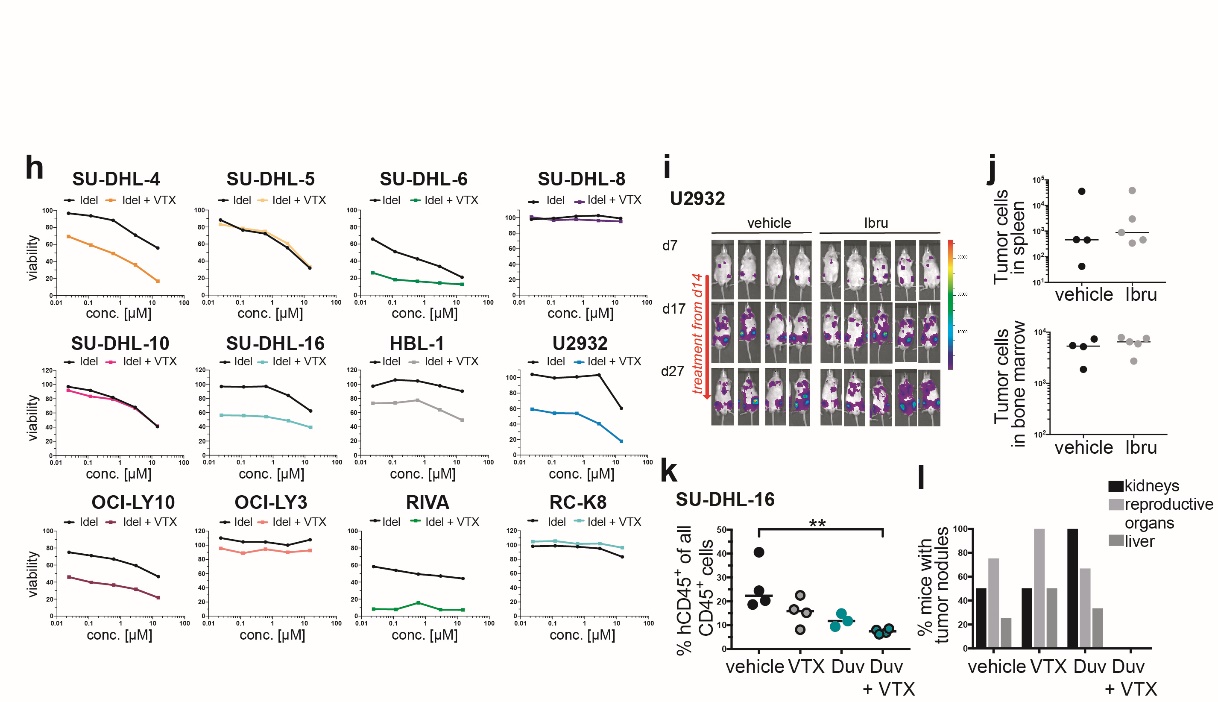
**

**Suppl. Figure 3.** **BTK and PI3K inhibition synergize with Bcl-2 inhibition to kill DLBCL cells of both cell-of-origin subtypes. a** Heat map displaying the viability (calculated as the mean of the five concentrations assessed per drug) of 13 DLBCL cell lines, as determined by CellTiter-Glo viability assay, after 48 hours of exposure to 65 manually selected indicated compounds, with or without additional exposure to 40 nM venetoclax. Five concentrations, generated by serial (four-fold) dilution, were tested per compound. See suppl. table 3 for the compounds and their respective highest concentrations. **b** Viability curves, derived from the drug screen, of the indicated cell lines, showing all five assessed concentrations of ibrutinib, either alone or in combination with venetoclax. The red cross indicates the viability upon exposure to 40 nM venetoclax as single agent. **c-f** Viability and synergy score heatmaps showing the mean viability (in %) of two independent experiments, conducted on the four indicated cell lines, upon 48 hours of exposure to venetoclax and ibrutinib at the indicated increasing concentrations. The Bliss and HSA methods were used to determine synergy. Scores >10 are indicative of synergy; scores < -10 indicate antagonism; scores ranging from 1-10 indicate additivity. **g,h** Validation of viability after 48 hours of exposure to duvelisib alone or in combination with venetoclax (g), or idelalisib alone or in combination with venetoclax (h) as assessed individually for each indicated cell line by CellTiter-Blue assay. A representative experiment of two independent ones per cell line is shown. **i,j** MISTRG6 mice were injected intravenously with 1 × 10^7^ U2932 cells; IVIS images were recorded once weekly (i). Mice received 25 mg/kg ibrutinib via oral gavage five times per week, initiated once lymphomas were clearly detectable in all mice of the cohort (after two weeks of growth). The lymphoma burden was quantified in the spleen (upper panel, j) and bone marrow (lower panel, j) at the study endpoint by flow cytometric staining for human (hCD45) and mouse CD45 (4 weeks post injection); differences were not statistically significant. **k,l** MISTRG mice were injected intravenously with 1 × 10^7^ SU-DHL-16 cells; mice received twice-weekly doses of 40 mg/kg venetoclax, either alone or in combination with 25 mg/kg duvelisib via oral gavage, starting after one week of growth. The lymphoma burden was quantified in the bone marrow at the study endpoint (four weeks post injection) by flow cytometric staining for human (hCD45) and mouse CD45 and by macroscopic examination of the indicated organs; only statistically significant differences are indicated in k; ** p<0.01.

**Suppl. Figure 4**

**
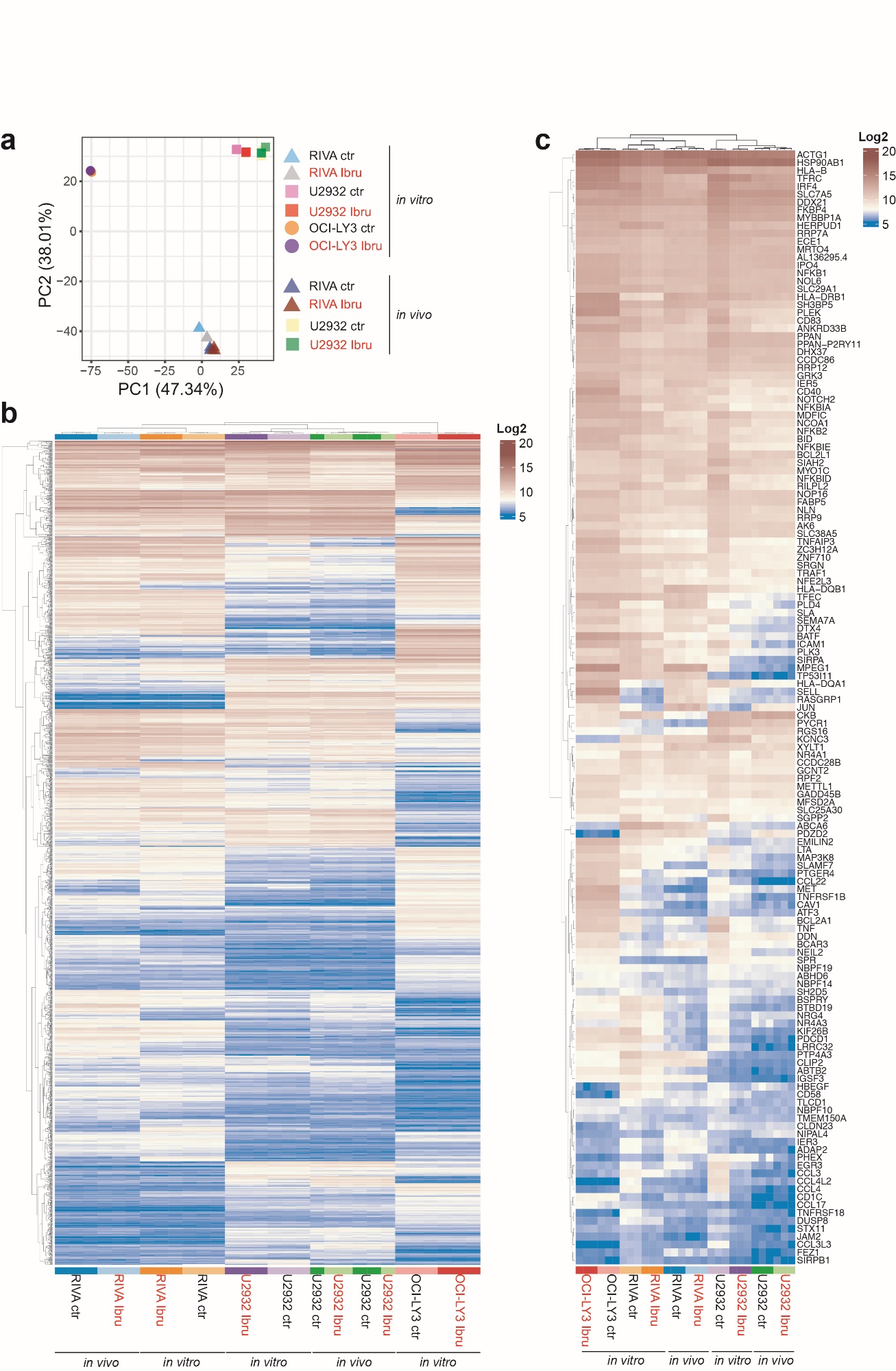
**

**Suppl. Figure 4. RNA sequencing reveals gene expression changes upon ibrutinib exposure *in vitro* and *in vivo*. a-c** RIVA, U2932 and OCI-LY3 cells were cultured in the presence or absence of ibrutinib for 6h and subjected to RNA isolation; U2932 and RIVA cells were additionally orthotopically transplanted into six MISTRG mice per cell line and subjected to two weeks of continuous *in vivo* ibrutinib exposure (25 mg/kg, administered five times per week). Bone marrow cells were harvested from three ibrutinib-treated and three vehicle-treated control mice, and tumor cells were isolated by cell sorting prior to RNA isolation. *In vitro* and *in vivo* generated triplicate samples were subjected to bulk RNA sequencing. **a** Principal component analysis based on all expressed genes segregates samples based on cell line identity first, *in vivo* vs. *in vitro* treatment second, and ibrutinib exposure last. **b** Unsupervised hierarchical clustering, displayed as a heatmap, of all 2000 differentially expressed genes across samples confirms the sample segregation as described in a. **c** Heatmap of 141 genes that are downregulated (log2 fold change <-0.5 and adjusted p-value <0.05) upon ibrutinib exposure *in vitro* in both RIVA and U2932 cells, shown for all 30 samples with all genes annotated.

**Suppl. Figure 5**

**
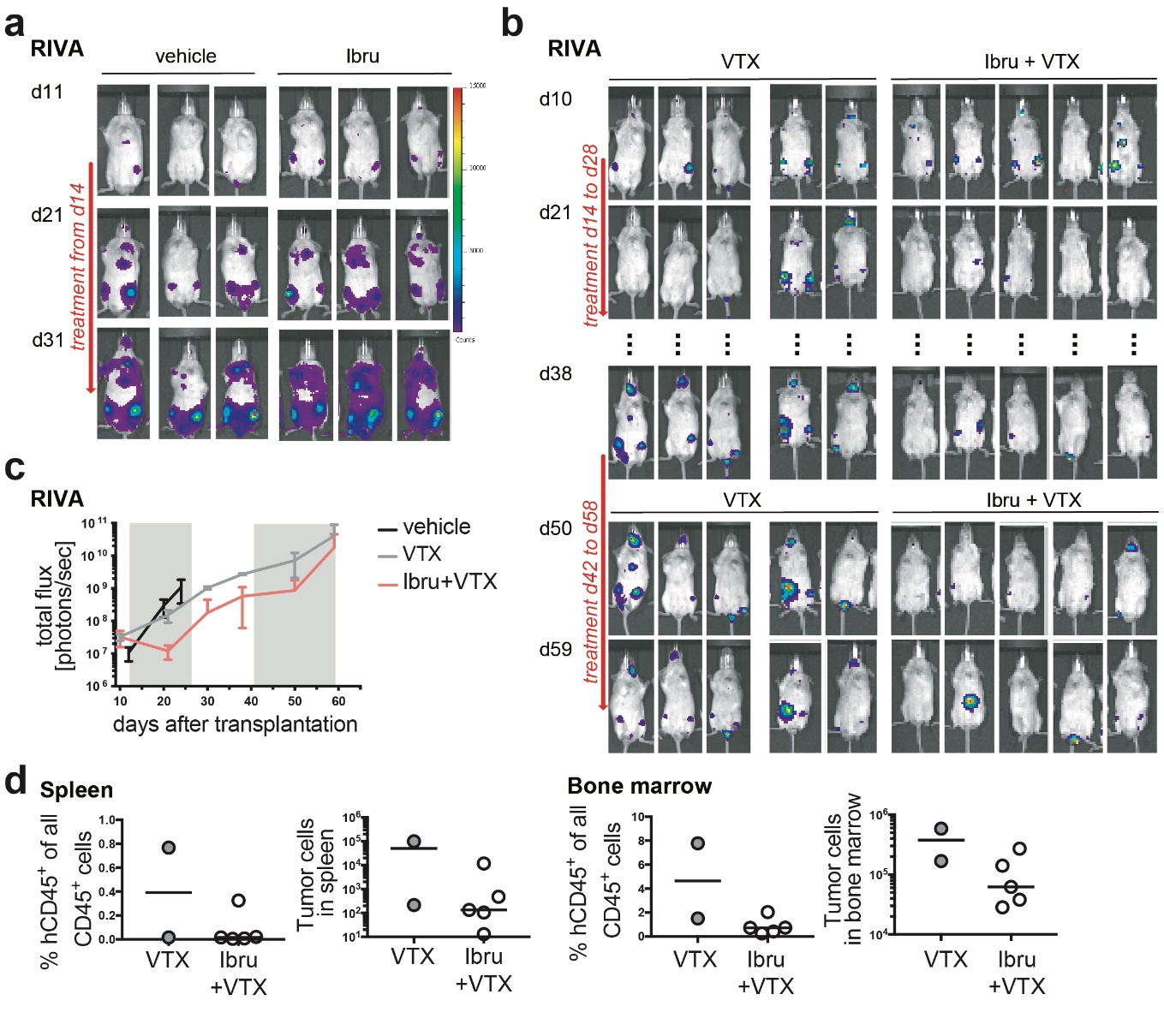
**

**Suppl. Figure 5. Acquired venetoclax resistance can be overcome by ibrutinib addition *in vivo*. a** MISTRG mice were injected intravenously with 1 × 10^7^ RIVA cells; IVIS images were recorded on the indicated days. Mice received five doses of 25 mg/kg ibrutinib per week via oral gavage, initiated once lymphomas were clearly detectable in all mice of the cohort (after two weeks of growth). **b-d** MISTRG mice were injected intravenously with 1 × 10^7^ RIVA cells; IVIS images were recorded on the indicated days (b) and the radiance was plotted longitudinally as means +/- SD for all treatment groups (c). Mice received twice-weekly doses of 40 mg/kg venetoclax, either alone or in combination with 25 mg/kg ibrutinib via oral gavage, initiated once lymphomas were clearly detectable in all mice of the cohort (after two weeks of growth; indicated by grey shading in c). After an interval of two weeks without drug treatment leading to lymphoma recurrence in a subset of mice, single and combination treatments were re-initiated for another two weeks. At the study endpoint at 9 weeks post injection, tumor cells were immunomagnetically sorted from several donors (the three mice in the left panel and two additional mice) for *ex vivo* resistance testing and re-transplantation (shown in main Figure 7), and the remaining mice were subjected to the quantification of their lymphoma burden in the spleen and bone marrow at the study endpoint by flow cytometric staining for human CD45 (hCD45) and mouse CD45 (d); differences in d are not statistically evaluable due to small sample size.

**Suppl. Figure 6**

**
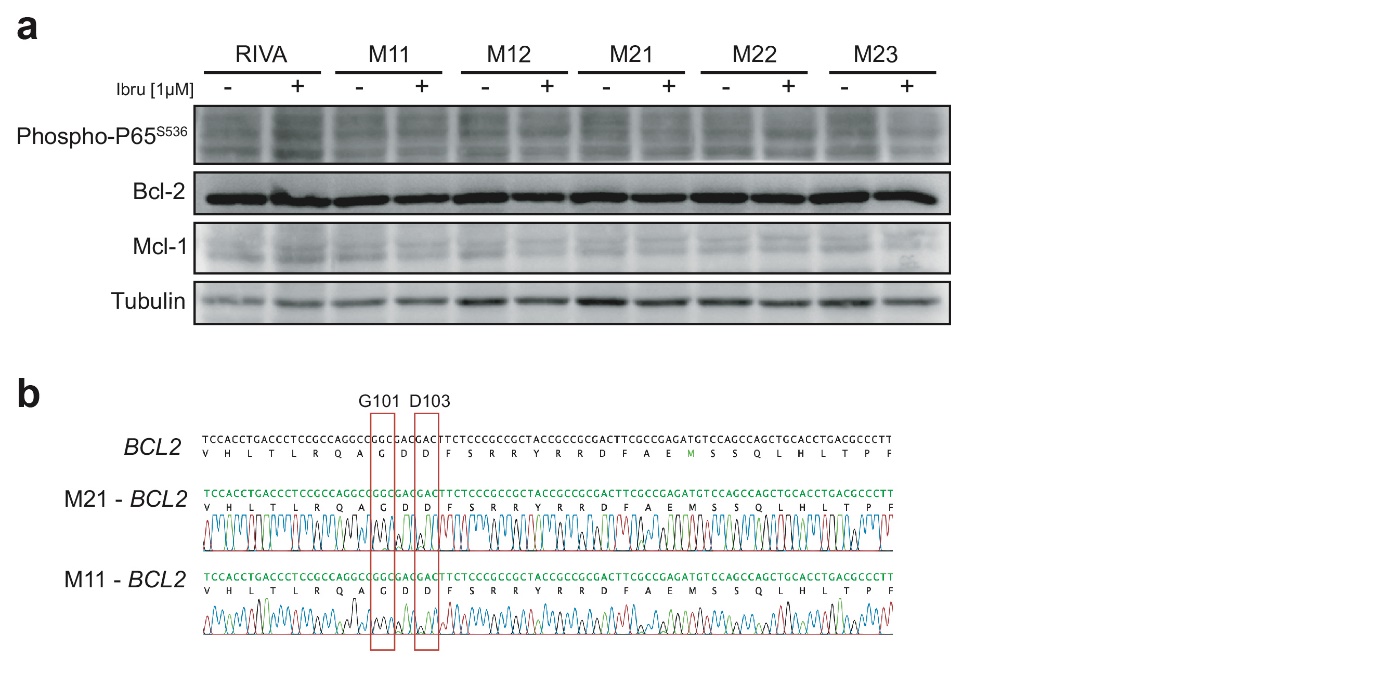
**

**Suppl. Figure 6. Venetoclax-resistant RIVA cells isolated from refractory mice do not differ from the parental cell line in terms of Bcl-2 or Mcl-1 expression or P65 phosphorylation, and have not accumulated mutations in their venetoclax binding site in Bcl-2. a** Expression of Mcl-1 and of Bcl-2 of the indicated RIVA re-isolates and parental cell line exposed or not to 1 μM ibrutinib for 48 hours, and phosphorylation of P65, as determined by Western blotting. **b** Sequence of the *BCL2* genomic locus at exon 1 harboring the venetoclax binding site. All examined clones show the wild type *BCL2* sequence.

**Supplemental materials and methods**

**Cell culture experimentation, drug screening and viability assays.** We used a panel of 13 DLBCL cell lines including six of the GCB subtype (SU-DHL-4, SU-DHL-5, SU-DHL-6, SU-DHL-8, SU-DHL-10, and SU-DHL-16), six of the ABC subtype (HBL-1, OCI-LY3, OCI-LY10, SU-DHL2, RIVA and U-2932) and one unclassified DLBCL cell line (RC-K8) that have all been previously described.[^1-3^](#_ENREF_1) Additional cell lines used for drug screening, but no other applications included the four Burkitt lymphoma cell lines BL60, Nawalma, Raji and Seraphine and the three mantle cell lymphoma cell lines HBL2, Maver and Mino. All cell lines were routinely tested, and negative for mycoplasma. Cell line authentication was performed as described.[^4^](#_ENREF_4) Cell lines were maintained at 37°C and 5% CO_2_ in IMDM (OCI-Ly3, OCI-LY10, RIVA) or RPMI (all others) supplemented with 10% (OCI-LY3, RIVA) or 20% (all others) heat-inactivated FCS and 100 U/mL penicillin and 100 µg/mL streptomycin. Apoptosis rates were determined by TMRE staining using 400nM TMRE followed by flow cytometry. Cell viability was assessed using the Cell Titer Blue metabolic activity assay (Promega) by 2.5 hour incubation with reaction mix. For drug response profiling, cell lines were seeded in 384-well plates at 2x10^5^ cells/mL. The cells were exposed to five different five-fold dilutions of the small-molecule inhibitors listed in suppl. table 1; suitable drug concentration ranges were individually chosen for each drug based on IC50 values determined in biochemical assays, manufacturer recommendations and the existing literature. For the venetoclax combinatorial drug screen, cells were exposed to five different four-fold dilutions of the small-molecule inhibitors listed in suppl. table 3 in the presence or absence of 40nM venetoclax (Selleckchem). Drug treatment was performed for 48 hours at 37°C and the viability was assessed by CellTiter-Glo assay (Promega) in both screens. The raw viability data from both screens is presented in suppl. tables 2 and 4; all other raw data from confirmatory viability assays are available from the authors upon request. Procedures for lentiviral gene transfer and genomic editing, for Western blotting, qRT-PCR and immunohistochemistry, RNAseq data analysis and TCGA data analysis are described in the supplemental methods.

**Animal experimentation and tissue processing.** M-CSFh;IL-3/GM-CSFh;hSIRPAtg;TPOh;Rag2-^-^γc- (MISTRG)[^5^](#_ENREF_5) and MISTRG mice that additionally express IL-6h (MISTRG6)[^6^](#_ENREF_6) were obtained from a local repository. For the orthotopic xenotransplantation model, cells were injected intravenously (1 × 10^7^ cells in 100 µl PBS) into 6- to 8- week-old mixed-gender MISTRG or MISTRG6 mice. The sample size depended on litter size. We aimed for samples sizes of 6 mice per group, which in this system is sufficient to produce significant results even if multiple comparisons corrections need to be applied. Animals were randomized into different treatment groups and of both genders. Spleens and bone marrow from hind legs were harvested for analysis at the study endpoint of orthotopic models. All animal studies were reviewed and approved by the Zurich Cantonal Veterinary Office (licenses 224/2014, 235/2015, 132/2019 and their amendments, to A.M.) Small molecule inhibitors were administered p.o. twice weekly with the following treatment dosages: venetoclax 40 mg/kg/day (in 5% DMSO, 50% PEG300, 5% Tween80, 40% ddH2O), ibrutinib 25 mg/kg/day (in 5% DMSO, 30% PEG300, 5% Tween80, 60% ddH2O), duvelisib 25 mg/kg/day (in 10% DMSO, 40% PEG300, 5% Tween80, 40% PBS). For patient‐derived xenograft transplantation, primary DLBCL cells (obtained from the Clinic of Hematology‐Oncology at the University of Zürich) were transplanted by i.v. injection (1 × 10^6^ cells) to MISTRG6 mice as previously described.[^1^](#_ENREF_1) Ethical approval for work with primary DLBCL cells was obtained from the Ethical Commission of the Canton of Zurich (KEK-ZH-Nr. 2009-0062/1).

**Lentiviral gene transfer and genomic editing.** Luciferase expressing DLBCL cell lines were generated as described previously.[^1^](#_ENREF_1) For CRISPR/Cas9-mediated gene-editing the DLBCL cell lines were lentivirally transduced to express Cas9 (pKLV2-EF1a-Cas9Bsd-W, Addgene #68343) and selected with Blasticidin S (ThermoFisher Scientific) for two weeks. In a second step, specific sgRNAs were lentivirally delivered (pKLV2-U6gRNA(BbsI)-PGKpuro2ABFP-W, Addgene #67974) to Cas9-expressing DLBCL cell lines using the following sgRNAs: Bcl-2 (BCL2.1: GACCTGACGCCCTTCACCGCG, BCL2.2: GAAAGCGTCCCCGCGCGG TGA), Akt-1 (AKT1.1: TGTGCCGCAAAAGGTCTTCA, AKT1.2: TCACGTTGGTCCACATCCTG), Akt-2 (AKT2.1: TCTCGTCTGGAGAATCCACG, AKT2.2: GCATCGAGAGGACCTTCCACG), Akt-3 (AKT3.1: AGAATGGACAG AAGCTATCC, AKT3.2: TAAGGTAAATCCACATCTTG), Rpa3 (RPA3.1: GTACGGGTTCCATCAACTCGA, RPA3.2: GGTTGGAAGAGTAACCGCCA) and a scramble control sgRNA (Scr: GAACAGTCGCGTTTGCGACT). Expression of sgRNAs (BFP expression) was analyzed five days after transduction by flow cytometry.

# Immunohistochemistry. DLBCL patient cohorts were stained for Bcl-2, Mcl-1 and P65 utilizing the following antibodies: Bcl-2, clone 124 from Ventana (Ventana 790-4464; Oro Valley, AZ, USA) prediluted, Mcl-1, sc-819 (polyclonal) from Santa Cruz (Santa Cruz, CA, USA) at a dilution of 1:100 and P65, ab31481 (polyclonal) from Abcam (Cambridge, UK) at a dilution of 1:20, respectively. Cut‐offs were determined as described previously,[^7^](#_ENREF_7)^,^[^8^](#_ENREF_8) and were set at >70% of tumor cells for Bcl-2, >30% for Mcl-1 and nuclear staining (corresponding to active/phosphorylated P65) in any tumor cell for P65. Informed consent to use archival material for scientific purposes was obtained from all subjects, and all experiments using human material conformed to the principles set out in the WMA Declaration of Helsinki and the Department of Health and Human Services Belmont Report.

**Western blotting.** Protein extracts were made in RIPA buffer (50 mM Tris-HCl, pH 8.0, 150 mM sodium chloride, 1% NP-40, 0.5% sodium deoxycholate, 0.1% SDS) supplemented with 2 mM sodium orthovanadate, 15 mM sodium pyrophosphate, 10 mM sodium fluoride, and 1× cOmplete protease inhibitor cocktail (Roche). Protein concentrations were determined by BCA assay (Pierce), and equal amounts were separated by SDS–PAGE using 10% acrylamide gels followed by transfer onto polyvinylidene difluoride membranes. Membranes were probed with antibodies (all from Cell Signaling) against PTEN (clone D4.3), Actin (clone 8H10D10), Tubulin (clone DM1A), AKT (clone C67E7), pAKT (clone T308), Mcl-1 (clone D35A5), Bcl-2 (clone D55G8), Bcl-X_L_ (clone 54H6), p-P65 (clone S536) and P65 (clone 8242P).

**Quantitive RT-PCR.** RNA was extracted using the NucleoSpin RNA mini kit (Macherey‐Nagel) according to the manufacturer’s instructions. Synthesis of complementary DNA was performed using Superscript III reverse transcription (Invitrogen) and TaqMan gene expression assays (Thermo Fisher Scientific) were performed for the following genes: BCL2L1 (Hs00236329_m1), BCL2A1 (Hs00187845_m1), MCL1 (Hs03043899_m1) and ACTB (Hs01060665_g1). The samples were analyzed on a LightCycler 480 instrument (Roche) and target mRNA abundance was subsequently calculated relative to beta-actin.

**TCGA RNA-Seq and whole exome sequencing analysis.** Gene-level counts of 481 samples were downloaded from the TCGA project NCICCR-DLBCL. Counts were generated by the STAR aligner[^9^](#_ENREF_9) and the human genome build GRCh38.p0. Clinical data associated with each sample was also downloaded from TCGA. Count files were imported into R version 4.0.2 for downstream analysis (including library normalization and transformation) in the DESeq2 package version 1.28.1. Samples that had a genetic subtype of “other” were excluded from downstream analysis, leaving a final count of 220 samples. The DESeq2 model incorporated only the genetic subtype classification. Variance stabilized transformed counts were used to generate boxplots of target gene expression as a function of genetic subtype. Global and pair-wise Kruskal-Wallis one-way analysis of variance was used to generate p-values of expression across and between genetic subtypes. Whole exome sequencing (WXS) BAM files of 472 patients were downloaded from the TCGA project NCICCR-DLBCL. BAM files were generated using the BWA aligner[^10^](#_ENREF_10) and the human genome build GRCh38.p0, and included marking of duplicate reads and base quality score recalibration following GATK best practice recommendations. Upon downloading, each BAM file was filtered using a BED file of a 258-gene pane, sorted, and indexed, using SAMtools.[^11^](#_ENREF_11) This panel was derived from the genes with variants as published in Schmitz et al.[^12^](#_ENREF_12) Due to a lack of matched normals, somatic variants were called using Mutect2 from GATK version 4.1.8.0 in tumor-only mode. Variants were then annotated using Funcotator from GATK. MAF files generated from Funcotator were imported into R using the R package Maftools version 2.4.05. Samples with a genetic subtype of “other” were excluded from downstream analysis, leaving a final count of 206 samples. Variants that had fewer than 10 reads supporting the variant allele, and variants that had an allele frequency of greater than 2% in the gnomAD exome database, were discarded. Oncoplots, co-oncoplots, and forest plots were generated using the respective Maftools function. For Bcl-2, Mcl-1 and Bcl-X_L_ gene expression, WXS samples were binned into four groups determined by their expression counts at 25% intervals.

**RNAseq.** RIVA, U-2932 and OCI-LY3 were treated for 6 hours with 1 µM ibrutinib (Selleckchem) or dimethyl sulfoxide (DMSO). RIVA and U-2932 were orthotopically transplanted into MISTRG mice and mice were treated for two weeks with ibrutinib (in 5% DMSO, 30% PEG300, 5% Tween80, 60% ddH2O; 25 mg/kg/day; 5 treatments per week) or vehicle starting from day 14 post transplantation. Tumor cells were isolated from bone marrow from hind legs by cell sorting (live human CD45^+^ cells) using a FACSAria III. RNA sequencing was performed by the Functional Genomics Center Zürich. RNA was extracted using the NucleoSpin RNA mini kit (Macherey-Nagel) following the manufacturer’s protocol. Extracted RNA was prepared for sequencing using the Illumina TruSeq Stranded mRNA Library Prep assay following the manufacturer’s protocol. Sequencing was performed on the Illumina NovaSeq 6000 using the S1 Reagent Kit v1.5 (100 cycles) as per manufacturer’s protocol. Demultiplexing was performed using the Illumina bcl2fastq Conversion Software. Individual library sizes ranged from 27.1 million to 40 million reads. RNA sequencing analysis was performed using the SUSHI framework,[^13^](#_ENREF_13) which encompassed the following steps: read quality was inspected using FastQC, and sequencing adaptors removed using fastp; alignment of the RNA-Seq reads using the STAR aligner[^9^](#_ENREF_9) and with the Ensembl human genome build GRCh38 (patch 10, Release 91) as the reference;[^14^](#_ENREF_14) the counting of gene-level expression values using the ‘featureCounts’ function of the R package Rsubread;[^15^](#_ENREF_15) differential expression using the generalized linear model as implemented by the DESeq2 Bioconductor R package;[^16^](#_ENREF_16) and Gene Ontology (GO) term pathway analysis using the hypergeometric over-representation test *via* the ‘enrichGO’ function of the clusterProfiler Bioconductor R package.[^17^](#_ENREF_17) All R functions were executed on R version 4.0.3 and Bioconductor version 3.10. Heatmaps and upset plots were generated using the Bioconductor R package ‘ComplexHeatmap’.[^18^](#_ENREF_18) Heatmaps, boxplots, and scatterplots of gene expression counts were generated using counts transformed using the `varianceStabilizingTransformation` function of DESeq2. All raw RNA sequencing data are available from GEO through the accession number GSE171763.

1. Hashwah H, Bertram K, Stirm K, Stelling A, Wu CT, Kasser S*, et al.* The IL-6 signaling complex is a critical driver, negative prognostic factor, and therapeutic target in diffuse large B-cell lymphoma. *EMBO molecular medicine* 2019 Oct; **11**(10)**:** e10576.

2. Hashwah H, Schmid CA, Kasser S, Bertram K, Stelling A, Manz MG*, et al.* Inactivation of CREBBP expands the germinal center B cell compartment, down-regulates MHCII expression and promotes DLBCL growth. *Proceedings of the National Academy of Sciences of the United States of America* 2017 Sep 05; **114**(36)**:** 9701-9706.

3. Stelling A, Hashwah H, Bertram K, Manz MG, Tzankov A, Muller A. The tumor suppressive TGF-beta/SMAD1/S1PR2 signaling axis is recurrently inactivated in diffuse large B-cell lymphoma. *Blood* 2018 May 17; **131**(20)**:** 2235-2246.

4. Juskevicius D, Muller A, Hashwah H, Lundberg P, Tzankov A, Menter T. Characterization of the mutational profile of 11 diffuse large B-cell lymphoma cell lines. *Leukemia & lymphoma* 2018 Jul; **59**(7)**:** 1710-1716.

5. Rongvaux A, Willinger T, Martinek J, Strowig T, Gearty SV, Teichmann LL*, et al.* Development and function of human innate immune cells in a humanized mouse model. *Nature biotechnology* 2014 Apr; **32**(4)**:** 364-372.

6. Das R, Strowig T, Verma R, Koduru S, Hafemann A, Hopf S*, et al.* Microenvironment-dependent growth of preneoplastic and malignant plasma cells in humanized mice. *Nature medicine* 2016 Nov; **22**(11)**:** 1351-1357.

7. Menter T, Ernst M, Drachneris J, Dirnhofer S, Barghorn A, Went P*, et al.* Phenotype profiling of primary testicular diffuse large B-cell lymphomas. *Hematological oncology* 2014 Jun; **32**(2)**:** 72-81.

8. Wenzel SS, Grau M, Mavis C, Hailfinger S, Wolf A, Madle H*, et al.* MCL1 is deregulated in subgroups of diffuse large B-cell lymphoma. *Leukemia* 2013 Jun; **27**(6)**:** 1381-1390.

9. Dobin A, Davis CA, Schlesinger F, Drenkow J, Zaleski C, Jha S*, et al.* STAR: ultrafast universal RNA-seq aligner. *Bioinformatics* 2013 Jan 1; **29**(1)**:** 15-21.

10. Luo R, Wong T, Zhu J, Liu CM, Zhu X, Wu E*, et al.* SOAP3-dp: fast, accurate and sensitive GPU-based short read aligner. *PloS one* 2013; **8**(5)**:** e65632.

11. Li H, Handsaker B, Wysoker A, Fennell T, Ruan J, Homer N*, et al.* The Sequence Alignment/Map format and SAMtools. *Bioinformatics* 2009 Aug 15; **25**(16)**:** 2078-2079.

12. Schmitz R, Wright GW, Huang DW, Johnson CA, Phelan JD, Wang JQ*, et al.* Genetics and Pathogenesis of Diffuse Large B-Cell Lymphoma. *The New England journal of medicine* 2018 Apr 12; **378**(15)**:** 1396-1407.

13. Hatakeyama M, Opitz L, Russo G, Qi W, Schlapbach R, Rehrauer H. SUSHI: an exquisite recipe for fully documented, reproducible and reusable NGS data analysis. *BMC bioinformatics* 2016 Jun 2; **17**(1)**:** 228.

14. Zerbino DR, Achuthan P, Akanni W, Amode MR, Barrell D, Bhai J*, et al.* Ensembl 2018. *Nucleic acids research* 2018 Jan 4; **46**(D1)**:** D754-D761.

15. Liao Y, Smyth GK, Shi W. The Subread aligner: fast, accurate and scalable read mapping by seed-and-vote. *Nucleic acids research* 2013 May 1; **41**(10)**:** e108.

16. Love MI, Huber W, Anders S. Moderated estimation of fold change and dispersion for RNA-seq data with DESeq2. *Genome biology* 2014; **15**(12)**:** 550.

17. Yu G, Wang LG, Han Y, He QY. clusterProfiler: an R package for comparing biological themes among gene clusters. *Omics : a journal of integrative biology* 2012 May; **16**(5)**:** 284-287.

18. Gu Z, Eils R, Schlesner M. Complex heatmaps reveal patterns and correlations in multidimensional genomic data. *Bioinformatics* 2016 Sep 15; **32**(18)**:** 2847-2849.
